# PGPB administration influences the cross-talk and interactions of rhizosphere and endophyte microbial communities in sunflower

**DOI:** 10.1016/j.crmicr.2025.100478

**Published:** 2025-09-25

**Authors:** Chiara Braglia, Daniele Alberoni, Loredana Baffoni, Sergio Angeli, Diana Di Gioia

**Affiliations:** a Dipartimento di Scienze e Tecnologie Agro-Alimentari, University of Bologna, Viale Fanin 44, 40127, Bologna, Italy; b Faculty of Agricultural, Environmental and Food Sciences, Free University of Bozen-Bolzano, 39100 Bolzano, Italy

**Keywords:** *Helianthus annuus*, Beneficial bacteria, Microbial networks, Microbiome-plant genotipe interaction, Endophytic bacteria, Rhizosphere microbiota dynamics

## Abstract

Plant Growth Promoting Bacteria (PGPB) represent a promising strategy for sustainable agriculture, offering the potential to modulate plant-associated microbiomes and enhance crop resilience. This study evaluated the impact of three microbial consortia (among *Bacillus,* Lactobacillaceae and *Paenibacillus*) on the rhizosphere and root endophytic microbiota of two sunflower (*Helianthus annuus*) genotypes: a hybrid (LST907 - Olival) and an open-pollinated variety (Peredovick), over two consecutive growing seasons. High-throughput 16S rRNA amplicon sequencing revealed that PGPB inoculation induced significant modulation of microbial communities, with strong genotype- and year-specific responses. Inoculants selectively enriched key beneficial taxa, including Pseudomonadaceae, Streptomycetaceae, Lactobacillaceae, and Comamonadaceae, while depleting others such as Chitinophagaceae and Micromonosporaceae, suggesting a functional shift in microbiome composition. Correlation and co-occurrence across endosphere and rhizosphere niches revealed enhanced connectivity between taxa under inoculated conditions, with stronger and more variable associations observed in Peredovick. Remarkably, several microbial families showed consistent cross-niche associations across years, suggesting robust and reproducible inoculant effects. Principal Component Analysis confirmed clear structural shifts in both rhizosphere and endosphere communities, with specific microbial families acting as key drivers of treatment-related variance. These findings provide evidence that PGPB not only shape microbial diversity at the community level but also influence microbiome assembly dynamics in the root endosphere and rhizosphere niches. Moreover, the sunflower genotype plays a central role in mediating the magnitude and direction of these microbiome shifts. Overall, our results support the development of genotype-adapted microbial inoculants to modulate plant microbiomes with enhanced functional potential, pointing to a perspective for improving crop performance under sustainable management frameworks.

## Highlights

• PGPB reshaped rhizosphere and endosphere microbiota in a genotype-specific way.
• Beneficial taxa like Pseudomonadaceae and Lactobacillaceae were enriched.
• Stronger rhizo–endophytic microbial links emerged after PGPB treatments.
• Peredovick showed greater microbiome plasticity than the hybrid variety.
• Inoculants modulated microbial networks across seasons and root compartments.

## Graphical abstract

**Figure:**
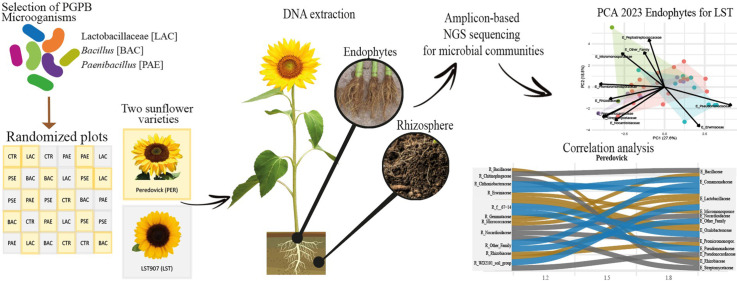

## 1 Introduction

Microorganisms play a fundamental role in maintaining the soil-ecosystem functionality by regulating biogeochemical cycles, driving the decomposition of organic matter, and mediating the fluxes of carbon, nitrogen, and other key elements (Basu et al., 2021; Raza et al., 2023; Zou et al., 2024). Among them, Plant Growth Promoting Bacteria (PGPB), abundant especially in the rhizosphere, contribute to the well-being and development of both cultivated and wild plants. They can establish symbiosis with plants, influencing plant growth, productivity and contributing to their health status (Fukami et al., 2016; Heinen et al. 2018). Among the different support functions exerted by PGPB nitrogen fixation, micro and macro elements stabilisation and solubilization, the induction of adaptive responses to environmental conditions is of fundamental importance (Piccin et al., 2013; Bender and van der Heijden, 2015; Fitzpatrick et al., 2020; Reis et al., 2020). The development of consortia capable of enhancing the positive plant-microorganism cross talk is a primary goal of sustainable crop management (Yusuf et al., 2025).

Although different plants are known to acquire target microbiomes, it has been shown that the geographical distribution and the pedo-climatic conditions (Haichar et al., 2008; Gottel et al., 2011; Massenssini et al., 2015; Adeleke et al., 2021), pollution, land use and other anthropic inputs (Tutu et al., 2012; McDaniel et al., 2014; Morgado et al., 2018; de Souza and Procópio, 2021) can shape the selective microbiome recruitment process. Moreover, plant genotype plays a key role in the selection and assembly of the associated microbiota (Aira et al., 2010; Shenton et al., 2016; Deng et al., 2021). In addition to plant genotype, temporal dynamics such as seasonal variation and interannual fluctuations also influence microbiota composition. It has been reported that microbial communities can shift significantly across seasons, reflecting changes of plant physiology, soil and environmental conditions both in perennial and annual crops (Wagner et al., 2016; Li et al., 2020). Moreover, in the same location, year-to-year variability in microbiota structure has been observed, suggesting that climatic and seasonal factors contribute to shaping the plant-associated microbial assemblages over time (Grady et al., 2019). These findings highlight the importance of considering both host genotype and temporal and seasonal shifts to fully understand the ecological dynamics and functional potential of targeted plant-associated microbiomes.

Sunflower (*Helianthus annuus* L.) is a major oilseed crop globally, valued not only for its economic importance but also for its adaptability to diverse environments. A wide range of both hybrid and non-hybrid sunflower varieties is available worldwide, with distinct agronomic and economic advantages depending on the production context (Vear, 2016). Specifically, hybrid varieties (e.g. modern cultivars, selected for high oleic acid production in the oil) are the result of controlled breeding programs between selected parent lines to enhance quantitative and qualitative characteristics exploiting the hybrid vigor (heterosis). In contrast, non-hybrid (open-pollinated varieties, without forced-pollination selection) varieties are genetically more diverse, are often used in low-input or organic farming systems, and its parental seeds can be used for subsequent harvests. Nowadays, sunflower hybrid varieties dominate the global production, due to the increased yield potential, the growth uniformity, and the enhanced tolerance to biotic and abiotic stresses rather than non-hybrid varieties (Seiler et al., 2017; Radanović et al., 2018). Moreover, the sunflower well-developed taproot system and its resilience in different environments make it a suitable model for studying plant–microbe interactions in the rhizosphere and endosphere. Consequently, in this study we compared a hybrid to a non-hybrid (open-pollinated) cultivar to assess whether the industrial genetic selection may influence the assembly and functional response of root-associated microbiota. Hybrid cultivars typically exhibit enhanced yield, uniformity, and stress resistance due to hybrid vigor (heterosis), whereas non-hybrid varieties maintain greater genetic diversity, potentially shaping microbial associations (Bueno de Mesquita et al., 2024). Such contrasting genetic structures may also lead to a diverse microbial response between the hybrid and the open-pollinated cultivar, showing a heterogeneous response across genotypes.

In the last decade, microbial-based strategies have been promoted and introduced on the market as bio-fertilizers in agriculture as an eco-friendly alternative to traditional chemical fertilizers (Bender et al., 2016). PGPB have been tested on a wide variety of crops, and their boosting effect on plant growth and resistance to stress factors has been validated on specific crops (Di Benedetto et al., 2017; Pardo-Díaz et al., 2021; Abdelaal et al., 2021). However, PGPB specificity of action has to be tested on the target crops, considering that the efficacy of administration and the plant response are not broad-spectrum for all cultivated plants. Sunflower is a high-demand oilseed crop requiring careful nutrient management to ensure optimal productivity and oil quality. The efficient use of macronutrients (N, P, K, S) and micronutrients (e.g., B, Zn) is essential for balanced vegetative growth, reproductive success, and stress resilience (Ali et al., 2013; Chaves et al., 2015; Belovolova et al., 2020). The use of PGPB may be helpful to mitigate the input costs and increase the improvement of sunflower crop management. Whereas some studies focused on the rhizosphere microbiota of sunflowers and on the factors that may influence its composition (e.g., genotype, soil type; Adeleke et al., 2021; Bueno de Mesquita et al., 2024), to the best of our knowledge, no studies have focused on PGPB administration and its effect on the autochthonous microbiota. Considering the importance of this crop for human consumption and its ecological importance (e.g., for pollinators support), the understanding of the potential of PGPB in the sunflower cultivation system is pivotal for the sustainability of its cultivation.

Therefore, in this work, we aim to investigate whether selected PGPB strains administered to the rhizosphere can influence the sunflower rhizosphere and the endophytic community. In addition, this study wants to elucidate whether different sunflower genotypes exhibit varying responses to the same microbial strains under identical environmental conditions. This is a critical aspect in understanding how the effectiveness of these new biofertilizers influence the rhizosphere dynamics, ultimately enhancing plant health and resilience, also in a holistic approach that considers microorganisms as active actors in the ecosystem interactions.

## 2 Materials & methods

About 40 microorganisms isolated from honeybee gut and pollen, rhizosphere and phyllosphere of different plant species were screened for their PGPB properties. Briefly, specific assays to determine manganese (MnO) and phosphate (PO_4_^3−^) solubilization, as well as indole-3-acetic acid (IAA) and siderophore production were carried out to select the best-performing microorganisms for field experiment. Moreover, an agar well diffusion assay was carried out to assess the antimicrobial activity against indicator strains. The protocols used for screening were fully reported in **Supplementary materials: Appendix 1**.

### 2.1 Selection of microbial strains, sunflower varieties, and *in field* experiment assessment

Among all the tested microorganisms, eleven strains were selected and massively cultivated in 2.5-liter flasks with the most appropriate growth media and growing conditions. Briefly, a total of 11 strains, among six *Bacillus* (BAC), three Lactobacillaceae (LAC) and two *Paenibacillus* (PAE), were selected for the field test (Table 1). *Bacillus* and *Paenibacillus* were incubated at 27 °C in agitation, while Lactobacillaceae at 37 °C in static microaerophilic conditions. All microbial strains were grown for 72 h obtaining an average concentration of 8.6 × 10^8^ CFU/ml, for BAC and PAE, and 8.2 × 10^8^ CFU for LAC/ml. The obtained PGPB were then diluted in water to reach the final concentration of approx. 5 × 10^6^ CFU and employed in the field.

**Table 1:** Strains used in the field, the relative abbreviation, and the experimental conditions established in this research.

Bacterial species used as PGPB	Strain	Abbreviation for the treatment group	Genotypes considered	
			LST 907 - Olival [LST]	Peredovick [PER]
			Experimental conditions	
*Bacillus aerius*	**SP12**	**BAC**	**BACLST**	**BACPER**
*Bacillus amyloliquefaciens*	**P5**			
*Bacillus amyloliquefaciens*	**SP43**			
*Bacillus licheniformis*	**SP9**			
*Bacillus toyonensis*	**SP27**			
*Bacillus subtilis*	**P17**			
*Apilactobacillus kunkeei*	**DAN39**	**LAC**	**LACLST**	**LACPER**
*Lactiplantibacillus plantarum*	**LB9**			
*Lactiplantibacillus plantarum*	**DAN91**			
*Paenibacillus humicus*	**SP29**	**PAE**	**PAELST**	**PAEPER**
*Paenibacillus alvei* S1	**PAA**			
No bacterial treatment	**–**	**CTR**	**CTRLST**	**CTRPER**

**Fig. 1:**
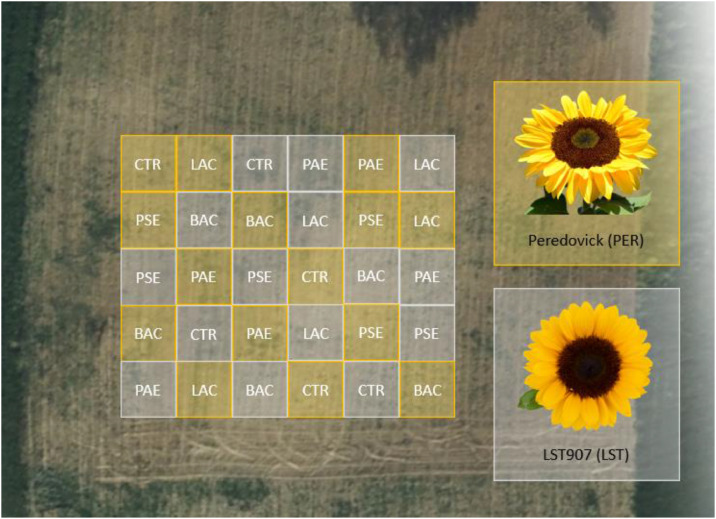
Experimental design and plant mapping for each experimental condition and the varieties tested.

Two commercial varieties of *H. annuus* were selected due to agronomical characteristics: PER the open-pollinated variety “Peredovick” (Arcoiris s.r.l., Modena, Italy) and LST a hybrid high-oleic variety “LST907-Olival” (Società Italiana Sementi, San Lazzaro di Savena, Bologna, Italy). The two varieties were chosen for their similar phenological developmental stages and plant size (verified in a pre-test, data not shown). Sunflowers were manually seeded in open field plots with a density of 5.5 plants per square meter (3 × 3 m plots - about 50 plants in total for each randomized plot, Fig. 1) in the Emilia-Romagna region (Castello di Serravalle, BO, 44°25′55.509′’N, 11°3′0.526′’E; 211 m a.s.l.). Plots were separated from each other by an empty zone of 1.5 m. Each mixture of microorganisms (BAC, LAC, PAE) was supplied in both sunflower varieties, PER and LST, combining between genotypes and different PGPB treatments (BACPER: *Bacillaceae* × Peredovick; LACPER: Lactobacillaceae × Peredovick; PAEPER: *Paenibacillus* × Peredovick; BACLST: *Bacillaceae* × LST; LACLST: Lactobacillaceae × LST907; PAELST: *Paenibacillus* × LST and finally the respective untreated controls: CTRPER and CTRLST). A total of four treatments were applied at the selected stages BBCH 0.01 (one week after the seeding), BBCH 1.14 (4^th^ leaf), BBCH 5.57 (flower about to bloom) and BBCH 6.65 (complete blooming) (Meier, 2001). Each microorganism was administered individually for each sunflower variety within irrigation water at the concentration of 5 × 10^6^ CFU/ml, for a total of 5L per plant (2.5 × 10^7^ CFU/plant). The same experimental design was carried out for both years, 2023 and 2024.

### 2.2 Rhizosphere and roots sampling and processing

Rhizosphere and root system were collected in each experimental condition at blooming time. Specifically, roots were carefully prepared as follows: a first washing with sterile saline solution (0.9 % NaCl) was performed to remove rhizosphere soil from the root system. The obtained rhizosphere pellet was then stored at –20 °C until microbial DNA extraction (Section 2.3). Moreover, to identify the endophytic microbial community, a second cycle of washings of the root system was performed with distilled water, followed by a wash with 70 % ethanol for 2 min, and two washes with a 3 % sodium hypochlorite solution for 5 min. A final washing step with distilled water was performed to remove possible traces of sodium hypochlorite that may degrade DNA during extraction. Sterile root samples were then lyophilized and stored at –80 °C until further use.

### 2.3 DNA extraction, genome sequencing and amplicon-based sequencing analysis

DNA extraction was performed using commercial DNeasy®PowerSoil® Pro Kit (Qiagen, Milan, Italy) for rhizosphere samples and DNeasy® Plant Mini Kit (Qiagen, Milan, Italy) for roots, according to the manufacturer protocols with some modifications. 250 mg of lyophilised roots were smashed using a mortar and steel pestle and homogenised with glass beads (0.01–0.1 μm) at 50 Hz in a rotovortex before beginning with the manufacturer's DNA extraction protocol. Fluorometric quantification of every sample was performed with Qubit Flex Fluorometer (Thermo Fisher Scientific, Milan, Italy), and DNA stored at –20 °C until further analysis. Finally, genomic DNA extraction from the selected PGPB strains was performed with Wizard® Genomic DNA Purification Kit (Promega, Milan, Italy). Whole genome sequencing was performed by MicrobesNG facility (Birmingham, UK) on IlluminaHI-Seq4000 platform. The amplicon-based sequencing for the rhizosphere and endophytic microbial communities was performed according to Baffoni et al. (2021), by IGA Technology Services (Udine, Italy) using specific primers for 16S rRNA V3-V4 region for both soil and root samples (**Supplementary Table S1**) relaying on PNA for chloroplasts exclusion from root samples (Beckers et al., 2016; Thijs et al., 2017).

### 2.4 Bioinformatic analysis

Raw reads were analysed with QIIME2 (Bolyen et al., 2019). The DADA2 plugin (Callahan et al., 2016) was used for paired-end reads joining, denoising and chimera removal. Samples with fewer than 10,000 reads were filtered out using the QIIME feature-table filter-samples command. Taxonomic assignment was performed using the QIIME feature-classifier classify-sklearn method, using the full-length sequences classifier of the Silva138 Database. Taxonomic composition at different levels and relative abundance plots were generated using the QIIME taxa barplot plugin. A rooted phylogenetic tree was constructed with the QIIME phylogeny align-to-tree-mafft-fasttree pipeline and used in the calculation of alpha and beta diversity metrics via the QIIME diversity core-metrics-phylogenetic plugin. Rarefaction curves were generated using the QIIME diversity alpha-rarefaction function. Diversity analyses were conducted on rarefied feature tables to account for differences in sequencing depth across samples. Alpha diversity was estimated using metrics such as Pilou Evenness index and Faith’s phylogenetic diversity. Beta diversity was calculated using both Weighted and Unweighted UniFrac indexes, as well as Bray–Curtis dissimilarity. Group comparisons were performed using Kruskal–Wallis tests to assess the effects of year, treatments and genotypes.

### 2.5 Data analysis

Statistical analysis on the whole dataset was conducted using R Studio (v. 4.3.3). Differential Abundance Analysis (DAA) of rhizosphere and endophytic bacterial families was assessed using a non-parametric approach based on Wilcoxon rank-sum tests. Data from 2023 to 2024 were analysed separately, only considering untreated sunflowers (CTRPER and CTRLST). Moreover, Dedicated analyses were carried out to compare the microbiome of sunflowers in the CTR group with each inoculated treatment, as well as to assess differences between the PER and LST microbial communities following BAC, LAC, and PAE administration. Average, standard deviation, and log_2_ fold change were performed, and Wilcoxon tests’ *p*-values were adjusted for multiple comparisons using the False Discovery Rate (FDR) method. Statistical analysis and plotting were performed using the ***tidyverse, ggplot2***, and ***writexl*** packages.

Principal Component Analysis (PCA) was performed separately for endophytic (E_) and rhizosphere (R_) microbial communities across two sunflower varieties (LST and PER) and two growing seasons (2023 and 2024). Prior to PCA, taxa with zero variance across all samples within each dataset were excluded to ensure dimensional stability. PCA was performed using the prcomp function, and results were visualised using ***ggplot2***. Convex hulls were used to highlight treatment-related clustering within the PCA space. At the same time, the top 10 contributing taxa were identified based on their loading vector norm, calculated as the Euclidean distance in Principal Components 1 and 2 (PC1-PC2). Only taxa with the highest contribution magnitudes were annotated and visualised as arrows (biplot vectors) to aid interpretation of microbial drivers behind sample separation. Statistical filtering and plotting were performed using the ***tidyverse*, *openxlsx***, and ***gridExtra*** packages.

To explore potential relationships between rhizosphere (R_) and endophytic (E_) microbial communities, pairwise Pearson correlations were calculated separately for each treatment and for each combination of plant variety (LST, PER) and year-specific responses (2023, 2024). Microbial families with an average relative abundance below 0.01 were excluded. Correlations were computed using the cor.test function and retained based on the following criteria: p 〈 0.05 for 2023 datasets, and *p* < 0.05 with |r| 〉 0.75 for 2024, to emphasize stronger associations in the more complex 2024 data. Data wrangling was performed using tidyverse package. Significant correlations were aggregated and visualized as Sankey plots with ***ggalluvial*** package, where rhizosphere and endophyte families appear as distinct layers, and flows indicate the strength of associations. Plots were built with ***ggplot2*** and arranged using ***patchwork***.

To highlight the strongest associations between endosphere and rhizosphere niches, co-occurrence networks were constructed from significant correlations (p 〈 0.01, |r| 〉 0.75). For each variety, the top 15 correlations were selected and visualized in circular layouts to emphasize taxonomic symmetry and niche origin. All data manipulation and filtering steps were performed using the ***dplyr*** and ***readr*** packages.

Rhizosphere microbial functional potential, following the PGPB administration, was inferred from community composition by mapping taxonomically resolved families that exhibited significant positive or negative shifts in the DAA analysis. The relevant families identified were linked to a limited set of specific PGP functions, such as phosphate solubilization, siderophore production, exopolysaccharide (EPS) and biofilm formation, ACC deaminase activity, nitrogen fixation, phytohormone metabolism (IAA and related pathways), cellulolytic capacity, and biocontrol/induced systemic resistance (ISR). For each experimental condition (genotype × year × treatment), data obtained from DAA, including both increased and decreased taxa, were combined with functional trait databases. Positive or negative functional contributions were estimated by integrating taxon abundance changes (log_2_ fold-change) with trait association scores, resulting in weighted functional profiles for each treatment. Accordingly, functional enrichment or reduction was interpreted as an inferred effect of treatment-driven microbial shifts, not as direct evidence obtained from functional assays.

## 3 Results

### 3.1 Description of PGPB activities of selected strains

Eleven microbial strains were used in this study for their assessment as potential plant growth promoters. Specifically, the strains were selected primarily based on their ability to solubilize MnO₂, PO₄³⁻, and producing siderophores, which we considered relevant for nutrient mobilization in soil. In addition, we also considered indolacetic acid (IAA) production and antimicrobial activity, as these traits are known to contribute to plant growth promotion and pathogen control. Considering the MnO_2_ and PO_4_^3−^ solubilizing strains, the three Lactobacillaceae strains showed the most promising results with a strong (+++) solubilization (*A. kunkeei* DAN39, *L. plantarum* DAN91 and *L. plantarum* LB9). IAA production ranged from 0.00 to 86.29 µg/ml, with the highest results observed for *Bacillus, Pseudomonas*, and *Stenotrophomonas* strains. *A. kunkeei* DAN39, *Sphingobacterium canadense* and *Comamonas* were the most performant strains regarding the siderophore production (+++). Finally, the antimicrobial peptides assay highlighted the strongest production in *Bacillus*, specifically in *B. licheniformis* SP9, *B. subtilis* F3, *B. amyloliquefaciens* SP43, and SP12. The complete results obtained for each tested strain were reported in the **Supplementary materials: Appendix 1**. However, considering the results of all the tests, the study was based on the most performant strains belonging to Lactobacill*aceae, Bacillus*, and *Paenibacillus.* Table 2 reports the chosen PGPB parameter results.

**Table 2:** PGPB activities of the selected microbial strains.

Microbial strain tested	MnO_2_ solubilization	PO_4_^3−^ solubilization	IAA production (µg/ml)	Siderophore	N° of indicator strains strongly inhibited in their growth
*Apilactobacillus kunkeei* DAN39	+++	+++	0.00	+++	0
*Lactiplantibacillus plantarum* DAN91	+++	+++	NA	-	0
*Lactiplantibacillus plantarum* LB9	+++	+++	0.00	-	0
*Bacillus aerius* SP12	-	+	10.22	-	11
*Bacillus amyloliquefacens* P5	-	+	6.15	-	7
*Bacillus amyloliquefacens* SP43	-	-	0.00	-	8
*Bacillus licheniformis* SP9	-	+	14.07	-	17
*Bacillus subtilis* P17	-	-	3.78	-	4
*Bacillus toyonensis* SP27	-	+	9.93	-	1
*Paenibacillus alvei* PA(A)	-	+	24.82	-	4
*Paenibacillus humicus* SP29	-	-	9.99	-	3

### 3.2 NGS results output

Raw paired-end reads (2 × 250 bp) were obtained from Illumina sequencing across all samples, providing a comprehensive overview of the bacterial communities associated with the experimental treatments for both rhizosphere and endophytes. After DADA2 filtering, a total of 5,458,949 sequences were retained for rhizosphere samples from 2023 (average 82,711 sequences per sample – the observed features rarefaction curves reached a plateau at approximately 16,000 sequencing reads), and 1,347,774 sequences for endophyte samples from 2023 (average 21,393 sequences per sample – plateau at about 5000 reads). For the 2024 dataset, a total of 2,587,596 sequences were retained for rhizosphere samples (averaging 36,965 per sample – with a plateau at approximately 10,000 reads) and 3,006,489 sequences for endophyte samples (averaging 41,756 per sample – with a plateau at approximately 4,000 reads). Ordination was visualized through Principal Coordinates Analysis (PCoA), and statistical differences among groups were assessed with PERMANOVA (adonis function, 999 permutations). Initial analyses conducted on the full dataset (combining both years) revealed a strong temporal effect (Pielou Evenness *p* < 0.01; Observed Features *p* < 0.01; Faith PD *p* < 0.05), with year-to-year variability dominating the microbial community structure and partially masking potential treatment effects (Bray-Curtis PCoA addressing the year-to-year samples clusterings is shown as Figure S1). Therefore, all diversity analyses were subsequently repeated for each year separately, allowing a more accurate assessment of treatment-specific responses within each growing season.

α- and β-diversity analyses conducted for each year (**Tables S2–S6**) indicated clear interannual differences in the sunflower rhizosphere microbiota, with shifts in both α- and β-diversity metrics when comparing 2023 and 2024 (**Table S2, Figure S1**). Pielou’s evenness (**Table S2, Figure S2**) revealed that in 2023 Lactobacillaceae inoculation decreased community evenness in LST compared with the control and the other microbial treatments, indicating a moderate dominance of some taxa (**Figure S1**). In contrast, in PER group, no consistent differences among treatmens have been detected. Faith’s phylogenetic diversity index was less responsive to inoculation but tended to decrease from 2023 to 2024 (*p* = 0.02 - **Table S3**), suggesting a contraction in the width of phylogenetic lineages represented across years.

**Fig. 2:**
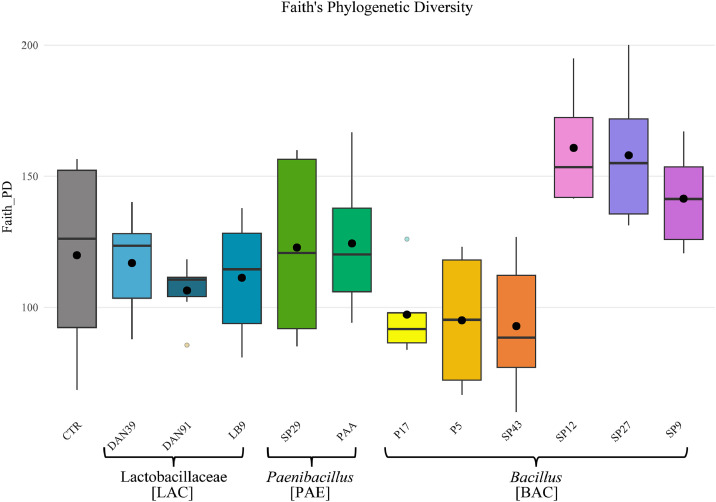
Faith’s Phylogenetic Diversity (Faith PD) index in sunflower rhizosphere soil samples collected in 2023. Boxplots represent the distribution of Faith PD across treatments: control (CTR), Lactobacillaceae strains (LAC: DAN39, DAN91, LB9), *Paenibacillus* strains (PAE: SP29, PAA, P17), and *Bacillus* strains (BAC: P5, SP43, SP12, SP27, SP9). Black dots indicate mean values.

When considering only 2023 (**Tables S7–S11**), inoculation effects were more evident. In LST, eveness showed a significant decrease in Lactobacillaceae treated group **(Figure S2** - **Table S7**) with a shift in community composition compared with the control, *Bacillus*, and *Paenibacillus*. Faith PD index, at strain Treatment level (Fig. 2**, Table S8**), varied significantly among treatments. Lactobacillaceae strains (DAN39, DAN91, LB9) consistently reduced Faith’s PD compared to the control, with DAN91 showing the most substantial reduction. *Paenibacillus* strains displayed contrasting effects, with SP29 maintaining high diversity and PAA and P17 markedly reducing it. *Bacillus* isolates generally sustained or increased diversity, particularly SP12 and SP27, which exhibited the highest Faith’s PD values among all treatments. These changes were supported by β-diversity metrics: both Unweighted and Weighted UniFrac (**Tables S9–S10**) revealed that Lactobacillaceae reshaped the phylogenetic structure and composition of the rhizosphere community, while Bray–Curtis dissimilarity (**Table S11**) highlighted marked differences in relative abundances. Together, these results indicate that inoculation in 2023 not only balanced species distribution but also altered both the presence/absence and relative abundance of taxa in LST. In contrast, PER displayed more heterogeneous responses, with some pairwise differences (e.g. control vs Lactobacillaceae) but without a consistent pattern across diversity indices. This variability likely reflects the genetic background of PER, an open-pollinated population, compared with the more uniform responses observed in the genetically homogeneous LST.

In 2024 (**Tables S12–S16**), diversity patterns were overall more homogeneous, and microbial inoculation did not result in significant changes in either evenness or phylogenetic diversity. β-diversity analyses confirmed that year-to-year variation contributed substantially to community structuring, whereas the effect of microbial treatments was less pronounced than in 2023.

### 3.3 H. annuus *plant genotype influences the rhizosphere and endophytic microbiome at the family level*

Rhizosphere (indicated as “*R_*” before taxa name) and endophytic (indicated as “*E_*” before taxa name) microbial communities were analysed both in the LST and in the Peredovick sunflower varieties, considering the whole set of experimental data obtained in the 2 seasons (2023 and 2024). The analysis of NGS data obtained was focused on the Phylum and family level of untreated sampled sunflowers (CTRLST and CTRPER), to assess the differences between genotypes unperturbed by microbial treatments.

In the rhizosphere, at the Phylum level, Bacillota emerged as the most dominant group in both varieties, with an average relative abundance of 31.1 % in LST and 29.2 % in PER. Pseudomonadota showed substantial presence, averaging 22.9 % in LST and 24.9 % in PER, while Actinomycetota was the third most abundant phylum, with 16.3 % in LST and 16.2 % in PER, reflecting a strong, consistent presence across sunflower varieties. Additional prominent phyla included Bacteroidota, Acidobacteriota, and Planctomycetota, with relative abundances of 10.9 %, 4.4 %, and 4.2 %, respectively, in LST, and 10.6 %, 4.6 %, and 4.3 % in PER. No significant statistical differences were detected in the phylum abundance between PER and LST. At family level, in the rhizospheres of LST and PER sunflower varieties, relevant differences were observed across several taxa. Within the Bacillota phylum, the Bacillaceae family showed an average abundance of 1.85 % in LST compared to 1.42 % in PER. In the Bacteroidota phylum, the most representative family, Bacteroidaceae, differed even less between the two varieties (3.3 % in PER vs. 3.2 % in LST). In both cases, the differences were not statistically significant. On the other hand, in the Actinomycetota phylum, Streptomycetaceae were significatively more abundant in PER, averaging 1.19 %, while in LST it was at 0.84 % (*p* < 0.05). Concerning families within the Pseudomonadota phylum, Pseudomonadaceae in LST reached an abundance of 5.26 %, while in PER this family was represented at 5.59 %. Rhizobiaceae showed a significantly higher abundance in PER (2.19 %) with respect to LST (1.76 %, *p* < 0.05). Similarly, Comamonadaceae varied, but without significance, with 0.90 % in LST and 1.23 % in PER. Within Bacillota, Lachnospiraceae was identified in PER at a relative abundance of 8.74 %, while it was at 9.02 % in LST. Clostridia_vadinBB60_group, another family within Bacillota, decreased in PER at 1.97 %, while the abundance increased 2.07 % in LST. Finally, in the Acidobacteriota phylum, families such as Vicinamibacteraceae were relatively similar, with 1.56 % in LST and 1.60 % in PER.

Considering the endophyte microbial community, the major phyla were represented by Pseudomonadota, Actinomycetota and Planctomycetota for both PER and LST. Specifically, Pseudomonadota exhibited significantly higher abundance in the LST variety (25.1 %) compared to PER (23.5 %), as well Planctomycetota (8.9 % in LST and 8.2 % in PER), and Actinomycetota (17.5 % in PER and 13.3 % in LST) without significant statistical relevance. However, a significant difference was observed just for Bacillota, more abundant in LST (3.8 %) than PER (2.5 %; *p* < 0.05). At family level, the most predominant taxa for both varieties were Pseudomonadaceae (12.5 % in LST and 13.3 % in PER). Considering each variety, LST was enriched in Erwiniaceae (6.2 %), Pseudonocardiaceae (6.03 %) and Rhizobiaceae (5.5 %), while PER in Pseudonocadioaceae (6.1 %), Streptomycetaceae (5.3 %) and Erwiniaceae (4.3 %). Among Pseudomonadota, Pseudomonadaceae were significantly enriched in PER (15.3 %) compared to LST (9.2 %; *p* < 0.01). Among Actinomycetota phylum, Streptomycetaceae were more abundant in PER (5.3 %) than in LST (4.4 %; p < 0.01), as well Nocardioidaceae was predominant in PER (4.3 %) respect LST (2.2 %, *p* < 0.01). Finally, within Bacillota, Lactobacillaceae showed an increase in PER (3.6 %) if compared to LST (1.9 %; p < 0.05). Data for both phyla and families sequenced in the rhizosphere and endophytes microbial communities are reported in **Supplementary file 1**.

**Fig. 3:**
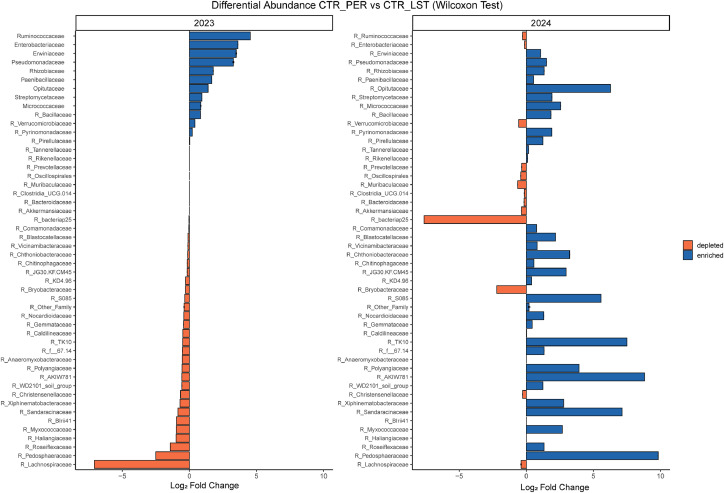
**Rhizosphere microbial community Differential Abundance Analysis (DAA).** Log fold change (LFC) values illustrate significant shifts in specific microbial taxa, with several microbial families demonstrating increased or depleted abundance in the PER variety relative to LST in the rhizosphere.

**Fig. 4:**
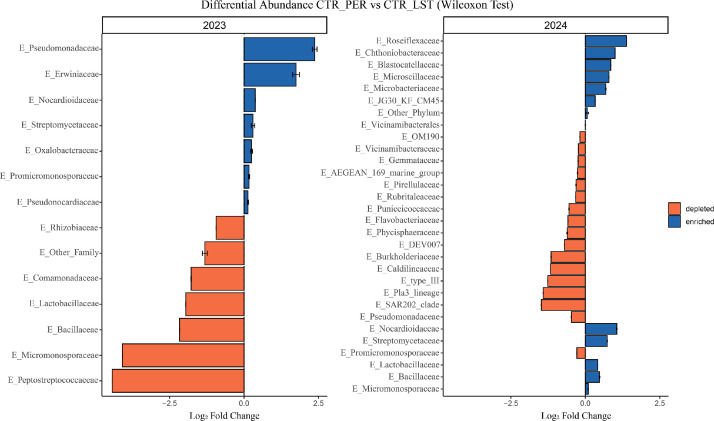
**Endophyte’s microbial community Differential Abundance Analysis (DAA).** Log fold change (LFC) values illustrate significant shifts in specific microbial taxa, with several microbial families demonstrating increased or depleted abundance in the PER variety relative to LST in the endophyte's community.

The differential abundance analysis (DAA) comparing the PER to the LST varieties (representing the control group in this study) provides insights into the single microbial families in the two years in both the rhizosphere (Fig. 3) and endophyte (Fig. 4) communities. Despite the differences obtained, no statistically significant variation was highlighted in the family microbial profile in both communities. However, in 2023 (Fig. 3**A**), LST showed a higher abundance of Lachnospiraceae, Pedosphaeraceae, and Roseiflexaceae compared to PER (LFC > 2.5), while PER rhizospheres were enriched in Ruminococcaceae, Enterobacteriaceae, Erwiniaceae, and Pseudomonadaceae (LFC > −2). In 2024 (Fig. 3**B**), PER showed an enrichment of Pedosphaeraceae, Sandaracinaceae, Opitutaceae, and underexplored taxa (e.g. AKIW781, TK10 and S085; LFC > 5), while LST was enriched in Bryobacteraceae and the unexplored taxa bacteriap25 (LFC > 2). Moreover, notable year-to-year shifts in the composition at family-level were detected. In both 2023 and 2024, comparisons between the varieties showed consistent enrichment and depletion patterns across several families. Most of the taxa (e.g. Pedosphaeraceae, Sandaracinaceae, Verrucomicrobiaceae, Ruminococcaceae, and unexplored taxa) followed an opposite trend when Peredovick and LST varieties were compared in the two years.

DAA on the endophyte microbial community highlighted differences between varieties and between the two years, as well. In 2023 (Fig. 4**A**), Peredovick was enriched in Pseudomonadaceae and Erwiniaceae (LFC > 2) and depleted in Peptostreptococcaceae, Micromonosporaceae, Bacillaceae and Lactobacillaceae (LFC < −2), if compared to LST. In 2024 (Fig. 4**B**), the enriched taxa in Peredovick were Roseiflexaceae, Nocardioidaceae, Chthoniobacteraceae and Streptomycetaceae (LFC > 0.7), while Burkholderiaceae, Caldilineaceae and underexplored taxa such as SAR202_clade and Pla3_lineage were depleted (LFC < −1.1), rather than LST. Finally, the endophyte community varied across years, also showing a higher number of microbial taxa in 2024. The comparison between varieties highlighted opposite trends of the same microbial taxa such as Pseudomonadaceae, Promicromonosporaceae, Lactobacillaceae, Bacillaceae and Micromonosporaceae, as reported for the rhizosphere community.

### 3.4 PGPB-induced reshaping of rhizosphere and endophyte microbial communities in sunflower varieties

A total of four treatments per season and for both years were carried out, during the specific phenological stages i) a week after the seeding (BBCH 0.01); ii) at 4^th^ leaf stage (BBCH 1.14); iii) flower stage near to bloom (BBCH 5.57); iv) and at complete blooming (BBCH 6.65). Roots and rhizosphere samples were carefully collected after the complete flowers ripening and senescence. To understand the possible influence of each microbial treatment on the composition and structure of the rhizosphere (R_) and endosphere (E_) of the varieties (LST and PER) and during the two growing seasons (2023 and 2024), Principal Component Analysis (PCA) was applied on the NGS absolute abundance results.

Principal Component Analysis (PCA) and Differential Abundance Analysis (DAA) revealed that PGPB treatments strongly influenced the composition of both rhizosphere and endophytic microbial communities, with clear genotype-, year-, and specific patterns for endosphere and rhizosphere niches (**Figures S5–S6**). The LST variety exhibited more pronounced shifts, especially in the rhizosphere in 2023 (PC1 = 43.2 %, **Figure S5B**) and the endosphere in 2024 (PC1 = 42.1 %, **Figures S5C**), indicating a strong responsiveness to inoculation. In contrast, the PER variety showed more subtle changes, with endosphere modulation being more evident in 2023 (**Figure S6A**), and both endosphere and rhizosphere niches structured in 2024 (**Figures S6C–S3D**). In particular, several key microbial taxa emerged as treatment-responsive indicators. For example, in LST endosphere (2023), Pseudonocardiaceae, Streptomycetaceae, and Nocardioidaceae contributed negatively to both principal components (**Figure S5A**), while in 2024, Blastocatellaceae, Chthoniobacteraceae, and Flavobacteriaceae showed strong positive loadings (**Figure S5C**), suggesting treatment-induced enrichment of potentially beneficial microbes. Similarly, in the LST rhizosphere (2023), taxa such as Pseudomonadaceae and Bacillaceae contributed negatively, whereas Gemmataceae and Vicinamibacteraceae were positively associated with treatment-related shifts (**Figure S5B**). Overall, the PCA results highlight clear influences of variety, year, and endosphere and rhizosphere niches on the sunflower-associated microbiota. A specific focus on the main shift highlighted in the PCA analysis is reported in **Supplementary materials: Appendix 2**. Moreover, the most PCA contributions significant taxa (*p* < 0.01) for each variety, endosphere and rhizosphere niches and season are reported in **Figure S7**, and reveal distinct microbial indicators associated with host genotype, soil environment, and inoculation treatments. The significant taxa and loadings that mainly contribute to PCA are reported in the **Supplementary file 1**.

Considering microbial enrichment or depletion between CTR and the microbial community after the PGPB inoculum, DAA revealed interesting results for both endophytes (**Figure S8**) and rhizosphere (**Figure S9**). In PER 2023 endophytes, BACPER **(Figure S8A**) enriched E_Enterobacteriaceae (LFC = 3.4, *p* < 0.01) and depleted E_Micromonosporaceae (LFC = −2.8, *p* < 0.01); LACPER (**Figure S8B**) increased E_Lactobacillaceae (LFC = 3.8, *p* < 0.01) and reduced E_Streptomycetaceae (LFC = −2.6, *p* < 0.01); PAEPER (Figure S4C) enhanced E_Comamonadaceae (LFC = 2.9, *p* < 0.01) and decreased E_Intrasporangiaceae (LFC = −3.1, *p* < 0.01). In 2024, BACPER (**Figure S8D**) promoted E_Pseudomonadaceae (LFC = 2.7, *p* < 0.01) and reduced E_Bacillaceae (LFC = −2.4, *p* < 0.01); LACPER (**Figure S8E**) enriched E_Lactobacillaceae (LFC = 3.5, *p* < 0.01) and depleted E_Xanthomonadaceae (LFC = −2.8, *p* < 0.01); PAEPER (**Figure S8F**) increased E_Streptomycetaceae (LFC = 2.6, *p* < 0.01) and decreased E_Chitinophagaceae (LFC = −3.0, *p* < 0.01).

In LST endophytes (2023), BACLST (**Figure S8G**) enriched E_Enterobacteriaceae (LFC = 2.9, *p* < 0.01) and depleted E_Sphingomonadaceae (LFC = −2.3, *p* < 0.01); LACLST (**Figure S8H**) increased E_Lactobacillaceae (LFC = 3.3, *p* < 0.01) and reduced E_Burkholderiaceae (LFC = −2.5, *p* < 0.01); PAELST (**Figure S8I**) enriched E_Comamonadaceae (LFC = 2.7, *p* < 0.01) and decreased E_Rhodobacteraceae (LFC = −2.6, *p* < 0.01). In 2024, BACLST (**Figure S8L**) promoted E_Pseudomonadaceae (LFC = 2.8, *p* < 0.01) and depleted E_Actinomycetaceae (LFC = −2.7, *p* < 0.01); LACLST (**Figure S8M**) enriched E_Lactobacillaceae (LFC = 3.2, *p* < 0.01) and reduced E_Micromonosporaceae (LFC = −2.9, *p* < 0.01); PAELST (**Figure S8N**) increased E_Streptomycetaceae (LFC = 3.0, *p* < 0.01) and decreased E_Bacillaceae (LFC = −2.8, *p* < 0.01).

Similarly, rhizosphere communities varied upon inoculation. In PER 2023, BACPER (**Figure S9A**) enriched R_Sphingobacteriaceae (LFC = 3.1, *p* < 0.01) and depleted R_Chitinophagaceae (LFC = −2.6, *p* < 0.01); LACPER (**Figure S9B**) promoted R_Lactobacillaceae (LFC = 3.5, *p* < 0.01) and reduced R_Burkholderiaceae (LFC = −2.3, *p* < 0.05); PAEPER (**Figure S9C**) increased R_Comamonadaceae (LFC = 2.9, *p* < 0.01) and decreased R_Rhodanobacteraceae (LFC = −2.8, *p* < 0.01). In 2024, BACPER (**Figure S9D**) enhanced R_Enterobacteriaceae (LFC = 2.8, *p* < 0.01), R_Pseudomonadaceae (LFC = 2.5, *p* < 0.01), and reduced R_Micromonosporaceae (LFC = −2.4, *p* < 0.01); LACPER (**Figure S9E**) enriched R_Lactobacillaceae (LFC = 3.2, *p* < 0.01) and decreased R_Sphingomonadaceae (LFC = −2.7, *p* < 0.05); PAEPER (**Figure S9F**) increased R_Streptomycetaceae (LFC = 2.6, *p* < 0.01) and reduced R_Rhodospirillaceae (LFC = −3.1, *p* < 0.01).

In LST rhizospheres (2023), BACLST (**Figure S9G**) enriched R_Pseudomonadaceae (LFC = 2.4, *p* < 0.01) and depleted R_Sphingomonadaceae (LFC = −2.9, *p* < 0.01); LACLST (**Figure S9H**) promoted R_Lactobacillaceae (LFC = 3.6, *p* < 0.001) and reduced R_Burkholderiaceae (LFC = −2.5, *p* < 0.01); PAELST (**Figure S9I**) increased R_Actinomycetaceae (LFC = 2.8, *p* = 0.006) and decreased R_Bacillaceae (LFC = −2.2, *p* < 0.05). In 2024, BACLST (**Figure S9L**) enriched R_Enterobacteriaceae (LFC = 2.7, *p* < 0.01) and depleted R_Micromonosporaceae (LFC = −2.3, *p* < 0.01); LACLST (**Figure S9M**) increased R_Xanthomonadaceae (LFC = 2.6, *p* < 0.01) and reduced R_Sphingobacteriaceae (LFC = −2.4, *p* < 0.05); PAELST (**Figure S9N**) enriched R_Streptomycetaceae (LFC = 3.1, *p* < 0.001) and depleted R_Chitinophagaceae (LFC = −2.9, *p* < 0.01). A specific focus on the DAA results among the same PGPB treatment during the two seasons (2023–2024) are reported in **Supplementary materials: Appendix 3**.

**Fig. 5:**
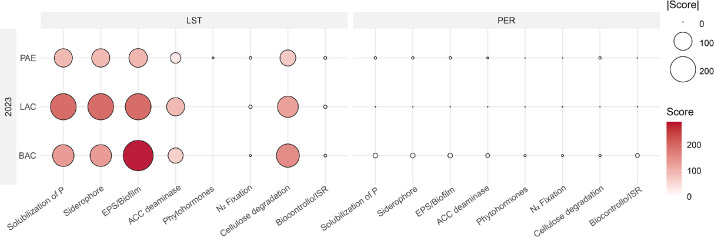
**Functional trait profiles of microbial families (2023) inferred from DAA analysis.** Functional trait profiles of microbial families showing significant variation in the DAA analysis across treatments (PAE, LAC, BAC) for the two sunflower varieties (LST and PER) in 2023. Circle size indicates the absolute trait association score (|Score|), while color intensity reflects the direction and magnitude of functional contributions (red = positive).

Inferred functional trait drivers highlighted marked variety- and treatment-specific patterns in the experiment carried out in the year 2023 (Fig. 5), but not for 2024. In the PER variety, the BAC treatment in 2023 was associated with the strongest positive modulation, particularly enhancing phosphate solubilization, siderophore production, and EPS/biofilm formation. These functions were primarily linked to Micrococcaceae and Comamonadaceae, which also contributed to ACC deaminase activity and potential biocontrol. Conversely, the PAE treatment in 2023 showed moderate negative contributions across the same functions, mainly associated with Streptomycetaceae. The LAC treatment in 2023 and all treatments in 2024 resulted in negligible or absent functional shifts in PER.

In contrast, the LST variety displayed pronounced functional enrichment in 2023 under both BAC and LAC treatments. These conditions stimulated phosphate solubilization, siderophore production, EPS/biofilm formation, and ACC deaminase activity, driven by multiple bacterial families including Pseudomonadaceae, Enterobacteriaceae, Bacillaceae, Micrococcaceae, and Paenibacillaceae. The PAE treatment also produced enrichment of similar functions, though to a lesser extent. In 2024, only BAC sustained mild functional enrichment, primarily linked to Pseudomonadaceae, while LAC and PAE had no detectable effects. Overall, LST showed consistent positive responses to treatments, whereas PER exhibited weaker or even negative responses, particularly under PAE treatments (See **Supplementary file 2**).

### 3.5 Correlation between rhizosphere and endophytes microbial communities

**Fig. 6:**
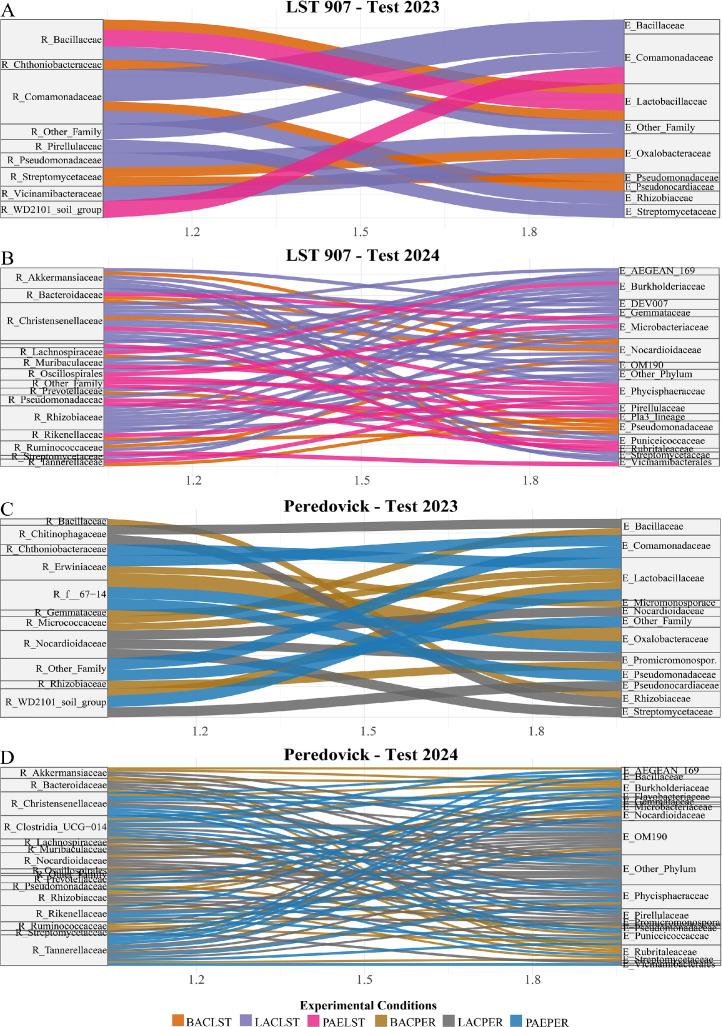
Sankey plots show rhizosphere taxa (R_) correlated with endophytes taxa (E_). A) Sunflower varieties LST907 tested in 2023; B) Sunflower varieties LST907 tested in 2024; C) Sunflower varieties PER tested in 2023; D) Sunflower varieties PER tested in 2024.

Correlation analysis between rhizospheric (R_) and endophytic (E_) bacterial families revealed consistent treatment-dependent interaction patterns across both LST and PER varieties in 2023 and 2024 (Fig. 6). In the LST 2023 (Fig. 6**A**), the control treatment (CTRLST) displayed a perfect positive correlation between R_Bacillaceae and E_Oxalobacteraceae (*r* = 1.00, *p* < 0.001). Within the BACLST treatment, R_Streptomycetaceae was significantly associated with E_Oxalobacteraceae (*r* = 0.64, *p* = 0.004). LACLST exhibited multiple strong correlations, including R_Comamonadaceae and R_Other_Family, which were both positively associated with E_Comamonadaceae (*r* = 0.85 and 0.80, respectively), and a negative correlation between R_Pseudomonadaceae and E_Oxalobacteraceae (*r* = –0.81, *p* = 0.008).

In PER 2023 (Fig. 6**B**), the BACPER treatment revealed a perfect negative correlation between R_Comamonadaceae and E_Flavobacteriaceae (*r* = –1.00, *p* = 0.002), and a strong positive association between R_Chitinophagaceae and E_Micromonosporaceae (*r* = 0.99, *p* = 0.004). LACPER showed two strong links between R_Verrucomicrobiaceae and E_Burkholderiaceae (*r* = 0.99, *p* = 0.001), and between R_Blastocatellaceae and E_Streptomycetaceae (*r* = 0.98, although non-significant (NS)). In PAEPER, R_Oxalobacteraceae was negatively correlated with E_Lactobacillaceae (*r* = –0.96, NS), while R_Gemmataceae was positively associated with E_Streptomycetaceae (*r* = 0.96, NS).

In LST 2024 (Fig. 6**C**), where only strong correlations (|r| > 0.75, *p* < 0.05) were considered, LACLST group, R_Oxalobacteraceae showed strong correlations with E_Streptomycetaceae and E_Gemmataceae (*r* = 0.84 and 0.83), while R_Bacillaceae were also linked to E_Gemmataceae (*r* = 0.82). In PER 2024 (Fig. 6**D**), BACPER, R_Blastocatellaceae and R_Chitinophagaceae were both strongly correlated with E_Micromonosporaceae (*r* = 0.95 and 0.94, respectively). PAEPER featured notable connections involving R_Chitinophagaceae and E_Burkholderiaceae (*r* = 0.93), as well as R_Comamonadaceae and E_Lactobacillaceae (*r* = 0.92).

Across all treatments and years, endophytic families such as Micromonosporaceae, Streptomycetaceae, Comamonadaceae, and Burkholderiaceae appeared as recurrent strong correlates of rhizospheric families, suggesting their possible role as microbial hubs mediating endosphere and rhizosphere niches interactions.

### 3.6 Recurrent and strongly correlated taxa between rhizosphere and endosphere communities

**Fig. 7:**
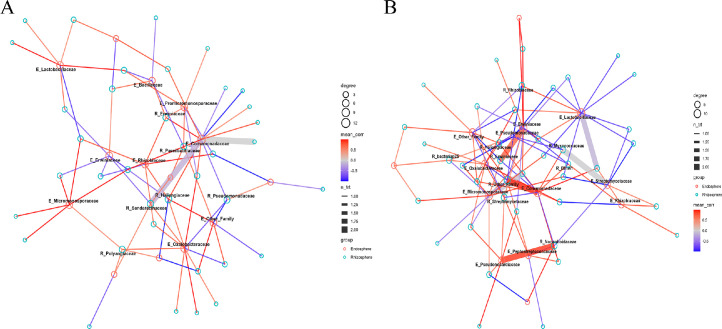
Strong correlation networks between rhizospheric and endophytic microbial communities in A) LST and B) PER varieties during year 2023. Networks report related taxa with *p* < 0.01.

As a preliminary approach to assess potential connections between the rhizosphere and endophytic communities, a simplified recurrent network analysis was performed. For this purpose, only strongly correlated taxa between R_ and E_ groups were included. Our results showed that the structure of strong correlation networks (*p* < 0.01) between rhizosphere and endophytic microbial families varied substantially across plant varieties and years (Fig. 7 and **Figure S10**). In 2023, both LST and PER varieties exhibited networks with a limited number of highly correlated associations (20 and 21 edges, respectively), characterized by elevated average correlation coefficients (mean *r* ≈ 0.58). The strongest associations for LST (2023) involved R_Bacillaceae and E_Oxalobacteraceae (*r* = 1.00, *p* = 0.0004), R_BIrii41 and E_Lactobacillaceae (*r* = 0.9999, *p* = 0.0035), and R_Haliangiaceae with E_Other_Family (*r* = 0.9999, *p* = 0.0056). For PER (2023), notable correlations were found between R_Rhizobiaceae and E_Nocardioidaceae (*r* = 0.9999, *p* = 0.0043), R_KD4–96 and E_Pseudonocardiaceae (*r* = 0.9697, *p* = 0.0014), and R_Pedosphaeraceae with E_Oxalobacteraceae (*r* = 0.9661, *p* = 0.0075). In contrast, the 2024 networks were denser, with 157 and 214 significant associations in LST and PER respectively yet showed markedly lower average correlation strengths (mean *r* = 0.074 for LST in 2024 and 0.229 for PER in 2024). Nevertheless, strong interactions persisted, such as between R_Oscillospirales and E_OM190 (*r* = 0.9999, *p* = 0.0013), R_Pyrinomonadaceae and E_Promicromonosporaceae (*r* = 0.9999, *p* = 0.0027), and R_Chitinophagaceae with E_Gemmataceae (*r* = 0.9999, *p* = 0.0031) in LST (2024). For PER (2024), the most prominent links included R_Akkermansiaceae and E_Burkholderiaceae (*r* = 0.9999, *p* = 0.0031), R_Tannerellaceae and E_Lactobacillaceae (*r* = 0.9999, *p* = 0.0065), and R_Micrococcaceae with E_Pseudomonadaceae (*r* = 0.9999, *p* = 0.0068).

**Fig. 8:**
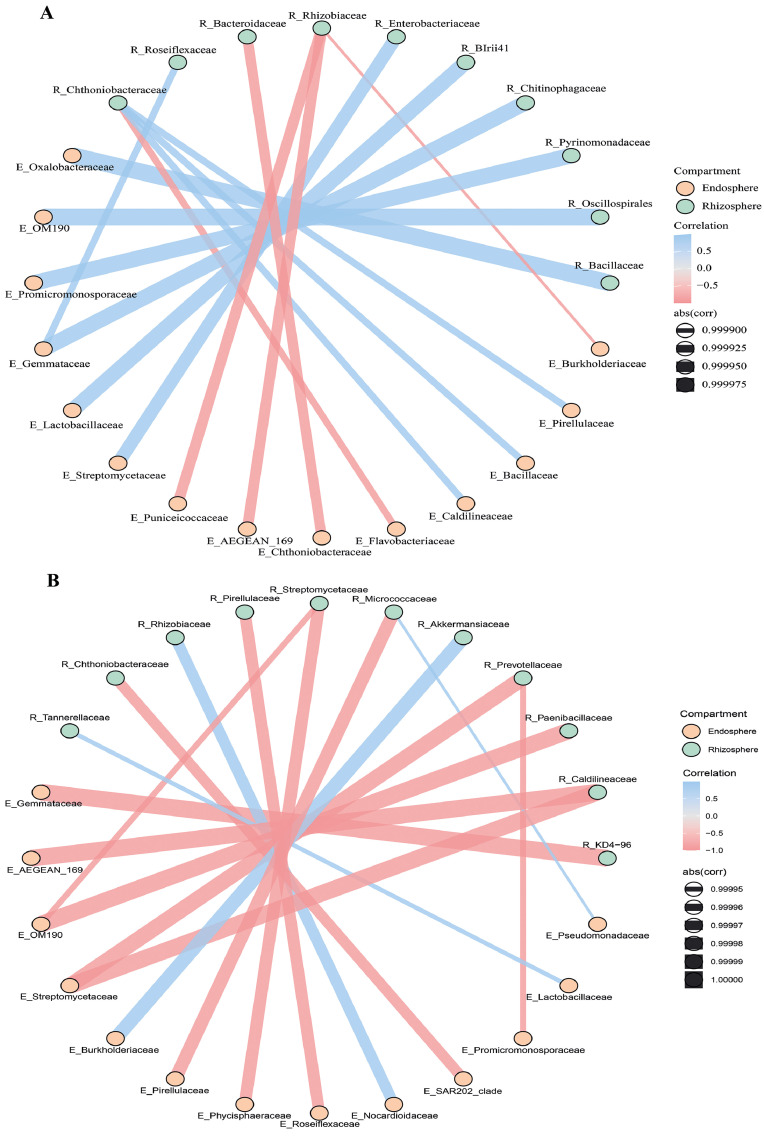
Circle network represents the top-15 most significant correlations (|r| > 0.75, *p* < 0.01) highlighted considering data globally (both test 2023 and test 2024 merged); A) LST907; B) PER.

Moreover, considering the whole merged dataset (2023 and 2024), a determination of the 15 more correlated taxa (|r| > 0.75, *p* < 0.01) between R_ and E_ was carried out. The microbial co-occurrence networks revealed distinct interaction patterns between rhizosphere and endophytic families in the two sunflower varieties (Fig. 8). In the LST network, associations were primarily driven by rhizosphere taxa such as R_Bacillaceae, R_Pyrinomonadaceae, and R_BIrii41, which displayed strong positive correlations with E_Oxalobacteraceae (*r* = 1.00, *p* = 0.0004), E_Promicromonosporaceae (*r* = 0.9999, *p* = 0.0027), and E_Lactobacillaceae (*r* = 0.9999, *p* = 0.0035), respectively. Notably, R_Oscillospirales exhibited a nearly perfect correlation with E_OM190 (*r* = 0.9999, *p* = 0.0013), suggesting a highly specific endosphere and rhizosphere niches association (Fig. 8**A**). In contrast, the PER network highlighted a different set of dominant interactions, including strong correlations between R_KD4–96 and E_Gemmataceae (*r* = 0.9697, *p* = 0.0014), R_Akkermansiaceae and E_Burkholderiaceae (*r* = 0.9999, *p* = 0.0031), and R_Streptomycetaceae with E_Streptomycetaceae (*r* = 0.9997, *p* = 0.0072) (Fig. 8**B**). Interestingly, reciprocal links between R_Pirellulaceae and E_Pirellulaceae were present in both LST and PER networks, suggesting a conserved interaction potentially reflecting shared functional roles.

## 4 Discussion

This study focused on the characterization of rhizosphere and root endophytic microbial communities associated with two sunflower varieties, LST907 and Peredovick, under different experimental treatments (administration of *Bacillus,* Lactobacillaceae and *Paenibacillus*), during two growing seasons.

The strains used in this study originated from diverse environments (pollen, bee gut, rhizosphere, etc.), and their ecological background may have shaped the observed outcomes. For instance, bee gut isolates, although not core members (as described by Motta and Moran 2024), may have acquired specific functions through interactions with adapted gut microbes, whereas strains from other sources could retain traits of their original habitat. This is consistent with our findings, where Lactobacillaceae (especially DAN39 and DAN91) reduced evenness, Faith’s PD, and intragroup variability, suggesting that members of this group may be particularly effective in selecting specific taxa, or in stimulating the plant to favour them.

The comparison of α- and β-diversity metrics across the detected year-specific responses, treatments, and genotypes highlights two main trends. First, inoculation effects were more pronounced in 2023, particularly in the LST genotype, where Lactobacillaceae treatments decreased community evenness, indicating an increase in dominance by a few taxa and a less balanced distribution of bacterial groups. This was accompanied by a clear shift in β-diversity, suggesting that inoculation altered both the taxonomic composition and the relative abundance of microbial lineages. By contrast, the PER population displayed more variable outcomes, with some differences among treatments but no consistent pattern across diversity indices. This heterogeneity can be attributed to the genetic background of PER, an open-pollinated population with a higher genotypic variability, which likely translates into less predictable rhizosphere responses to microbial inoculation compared with the more genetically uniform LST.

Second, the differences observed between 2023 and 2024 suggest that interannual variation has a substantial influence on rhizosphere community structure. While treatment effects were evident in 2023, diversity patterns appeared more homogeneous in 2024, with limited responses to inoculation. A possible explanation of these variations may be linked to interannual climatic variability, in particular extreme temperature shifts and extreme precipitation, that might significantly reshape soil microbial communities by altering moisture availability and biogeochemical processes (Shi et al., 2020; Lei et al., 2024) [please refer to the section “study limitations” in the supplementary materials]. Additionally, slight differences in seed-associated microbiota or genotype-specific traits, even among nominally identical lines, may influence early microbial assembly and contribute to year-to-year variability in community structure (Morales Moreira et al., 2021; Romão et al., 2025). However, this temporal effect overrode the detection of treatment-specific patterns when analysing the combined dataset (2023 and 2024). Accordingly, stratified analyses by year were conducted to disentangle treatment effects from overarching temporal variability and to improve resolution of treatment-associated community shifts.

At the phylum level, the sunflower rhizosphere is dominated by Bacillota, Pseudomonadota, Actinomycetota, Bacteroidota, Acidobacteriota, and Planctomycetota, confirming previous findings (Adeleke et al., 2021; Bueno de Mesquita et al., 2024). Relative abundances varied slightly across genotypes and endosphere and rhizosphere niches, where Pseudomonadota and Bacteroidota were more abundant in endophytes, whereas Acidobacteriota and Actinomycetota prevailed in rhizosphere samples (Adeleke et al., 2021). Saccharibacteria and Gemmatimonadetes, noted by Adeleke et al. (2021) in the endosphere, were less represented in our rhizosphere samples, highlighting a diverse endosphere -specificity. On the other hand, compared to Bueno de Mesquita et al. (2024), our data aligned but showed higher Bacillota abundance, probably due to edaphic or climatic variation. These dominant phyla have well-known ecological roles: Pseudomonadota support nutrient cycling and plant growth via nitrogen fixation and phytohormone production (Compant et al., 2010); Bacillota (e.g., *Bacillus* spp.) aid pathogen suppression and phosphate solubilization (Liu et al., 2020); Actinomycetota decompose organic matter and inhibit pathogens (Barka et al., 2016); Acidobacteriota are active in carbon cycling under oligotrophic conditions (Kielak et al., 2016); and Planctomycetota contribute to nitrogen cycling and complex matter degradation (Dedysh and Ivanova, 2019). These traits confirm a robust and functionally relevant core microbiome in the sunflower rhizosphere. Bacteroidota, with relative abundance near 10 %, appear functionally important and potentially influenced by genotype. This phylum contributes to phosphorus mobilization and nutrient cycling via phosphatase activity (Lidbury et al., 2021; Pan and Cai, 2023; Ling et al., 2022). Families like Chitinophagaceae, more abundant in PER, suggest enhanced polysaccharide degradation (Li et al., 2018), while low-abundance taxa may still provide specialized enzymatic functions (Larsbrink and McKee, 2020). These differences suggest variety-specific selection shaping Bacteroidota communities and their roles in plant–microbe interactions. Further genotype-specific differences were observed at the family level. PER showed higher levels of Bacillaceae, Micrococcaceae, Nocardioidaceae, Pseudomonadaceae, Lachnospiraceae, and Ruminococcaceae, as well as increased Erysipelotrichaceae, Enterobacteriaceae, and Alcaligenaceae, families usually linked to soil nutrient cycling (Wang et al., 2021; Li et al., 2024). Conversely, LST had a higher abundance of Thermomonosporaceae, Hungateiclostridiaceae, and Exiguobacteraceae, suggesting more active biomass degradation and anaerobic processes (Omori et al., 2019; Dobrzyński et al., 2021; Li et al., 2024). These findings highlight strong genotype-dependent microbial patterns, with each variety supporting distinct functional microbial assemblages.

Microbial inoculants induced consistent shifts in rhizosphere and endophytic communities across years and sunflower genotypes. In the rhizosphere, BAC treatments enriched Enterobacteriaceae and Pseudomonadaceae, families linked to nutrient cycling and growth promotion (Jha et al., 2011; Ramesh et al., 2014; Hu et al., 2017), while depleting Chitinophagaceae, Sphingobacteriaceae, and Micromonosporaceae, favoring fast-growing and nutrient-rich living taxa (Compant et al., 2019). LAC addition increased Lactobacillaceae, confirming inoculant persistence (Trivedi et al., 2020), often reducing Burkholderiaceae, suggesting competitive exclusion (Berendsen et al., 2012). PAE inoculant led to broader changes, enriching Streptomycetaceae, Comamonadaceae, and Actinomycetaceae, possibly due to antimicrobial activity. Endophytic communities responded more selectively but reflected rhizosphere trends. The repeated enrichment of Lactobacillaceae, Pseudomonadaceae, and Enterobacteriaceae across treatments suggests direct translocation (Hardoim et al., 2008) or plant-mediated selection (Trivedi et al., 2020). Concurrent depletion of Micromonosporaceae and Chitinophagaceae may reflect sensitivity to competition or micro-environmental shifts. Overall, these findings show that microbial treatments can reproducibly modulate plant microbiota, supporting their use in sustainable crop strategies (Backer et al., 2018).

PCA results confirmed clear structural differences between rhizosphere and endosphere microbial communities (Fig. S5 and S6). The rhizosphere consistently exhibited a higher variance explained by PC1, indicating a more dynamic and structured community, likely reflecting greater environmental responsiveness. In contrast, endosphere communities appeared more evenly distributed along the axes, suggesting stronger host-driven filtering. The endosphere and rhizosphere niche-specific pattern remained stable across years and sunflower genotype. Microbial treatments significantly influenced community composition, particularly in the rhizosphere, where the evident clustering in the PCA analysis highlight inoculant-driven restructuring. These changes support the role of microbial applications in modulating community assembly and enhancing plant–microbe interactions (Berg et al., 2021; Pardo-Díaz et al., 2021). In particular, taxa such as Vicinamibacteraceae, Gemmataceae, and Pseudomonadaceae in the rhizosphere, and Streptomycetaceae, Rhizobiaceae, and Promicromonosporaceae in the endosphere, showed the strongest significant contributions to PC1, suggesting they were particularly responsive to treatment. Given their known roles in nutrient cycling, antimicrobial compound production, and plant growth promotion (Santoyo et al., 2016; Berg et al., 2021; Srivastava et al., 2025), the response of these taxa points to a potential shift not only in community structure but also in microbiome functional capacity. The strong PC1 loadings for these groups highlight the ability of microbial inoculants to reconfigure plant-associated microbial networks both locally (in the rhizosphere) and systemically (within plant tissues) (Trivedi et al., 2020).

The correlation analysis between rhizosphere and endosphere microbial families revealed distinct and treatment-dependent patterns. Strong positive intra-family correlations, such as between Comamonadaceae in both endosphere and rhizosphere niches (ρ = 0.85), suggest potential direct microbial transfer or active selection by the host plant. Conversely, significant negative correlations, like that between Pseudomonadaceae (rhizosphere) and Oxalobacteraceae (endosphere), may indicate antagonistic interactions or competitive exclusion. These associations were often exclusive to bacteria-treated conditions and absent in controls, underscoring the influence of microbial treatments in modulating rhizosphere–endosphere connectivity and shaping microbial community assembly (Trivedi et al., 2020). Such patterns likely reflect host-mediated filtering mechanisms, where plants selectively recruit functionally compatible or phylogenetically related taxa, especially when the microbial environment is altered by inoculation (Trivedi et al., 2020). The involvement of beneficial families like Bacillaceae and Streptomycetaceae in strong positive correlations suggests the formation of cooperative microbial consortia promoting plant health (Timofeeva et al., 2023; Wu et al., 2023). These associations may resist environmental stresses or niche competition, favoring bacteria adapted to both soil and multi-host endophytic lifestyles. Significant correlations (p < 0.001) underline the strength of these interactions. In untreated conditions (CTRLST), some families showed near-perfect correlations (e.g., R_Bacillaceae – E_Oxalobacteraceae, *r* = 1.00), hinting at natural associations or co-selection. In contrast, inoculated treatments (e.g., BACLST) altered correlation profiles, such as R_Streptomycetaceae with E_Oxalobacteraceae, suggesting that microbial inputs can shift community dynamics and promote specific taxa. These results show that treatments affect not only individual niches (R_ or E_) but also cross-niche interactions, enhancing microbiome connectivity. This supports microorganism-based strategies to strengthen beneficial networks and boost crop performance (Chaparro et al., 2012; Bender et al., 2016; Negi et al., 2024). The microbial treatments applied in this study induced significant shifts in both rhizosphere and endophytic communities. Although, it is important to note that our analysis did not include direct functional validation. The strong positive and negative correlations observed among taxa in our study may reflect competitive or cooperative interactions that could shape microbial community stability and influence sunflower health. Strongest rhizosphere functional modulations were predicted in 2023 under the BAC treatment, and enhanced phosphate solubilization, siderophore production, and EPS/biofilm formation, mainly driven by Micrococcaceae and Comamonadaceae (Chhetri et al. 2022; Khalofah et al., 2021) might be hypotesized. Conversely, the PAE treatment in the same year may have led to a moderate reduction in these functions, again associated with the same families (Ma et al., 2023; Hansda et al., 2017). The LAC treatment in 2023 and all treatments in 2024 did not predict consistent shifts in PER. Conversely, the LST variety showed a markedly different trend. In 2023, both BAC and LAC treatments theoretically induced a strong enrichment of phosphate solubilization, siderophore production, and EPS/biofilm formation, with Pseudomonadaceae, Comamonadaceae, and Micrococcaceae as the main drivers. These taxa are well known for siderophore production, ACC deaminase activity, and IAA metabolism (Ghadamgahi et al. 2022; Preston 2004; Ma et al. 2023; Chang et al. 2024). The PAE treatment also promoted the same supposed functions, although to a lesser extent, while in 2024 only BAC produced mild enrichment, mostly supported by Pseudomonadaceae. Overall, BAC and LAC treatments in LST might stimulate nutrient mobilization and biofilm-related functions, while PER showed weaker and sometimes negative responses, particularly under PAE, consistent with previous reports on the strain-specific contribution of *Pseudomonas, Bacillus, Comamonas*, and *Arthrobacter* to PGP activities (Akinrinlola et al. 2018; Tsotetsi et al. 2022; Chhetri et al. 2022). While these interpretations remain speculative, our study provides a framework for understanding the potential ecological relevance of treatment-driven microbial shifts. Future studies combining community profiling with functional assays and plant performance data will be essential to validate these hypotheses.

Finally, cross-niche microbial correlations revealed strong, treatment- and variety-specific patterns linking rhizosphere and endosphere taxa. In both LST and PER, the top 15 correlations showed high value (ρ > 0.82), suggesting coordinated dynamics or niche exclusion across root endosphere and rhizosphere niches. PER exhibited a markedly higher number of strong correlations, especially under BAC and LAC treatments, pointing to a more plastic and responsive microbiome structure. To support this hypothesis our evidence includes Pseudomonadaceae and Promicromonosporaceae under BAC treatment, both associated with biocontrol and antibiotic production (Mercado-Blanco and Bakker, 2007; Barka et al., 2016), and Chitinophagaceae under LAC treatment, known for chitin degradation and potential antifungal activity (Hou et al., 2021; Costa et al., 2023). Under PAE treatment, correlations such as Burkholderiaceae–Caulobacteraceae may suggest synergistic functions in nitrogen fixation and biofilm formation (Poindexter, 1981; Estrada-De Los Santos et al., 2001). On the other hand, LST showed fewer but still strong correlations, with taxa like Verrucomicrobiaceae and Nocardioidaceae appearing under LAC and BAC treatments, indicating roles in polysaccharide breakdown and antimicrobial metabolite production (Barka et al., 2016). Moreover, key rhizosphere taxa such as Bacillaceae, Rhizobiaceae, and Pyrinomonadaceae were strongly linked with endophyte families such as Oxalobacteraceae, Lactobacillaceae, and Streptomycetaceae. These associations may reflect metabolic commensalism or recruitment pathways favoured by this variety (Abedini et al., 2021). Specifically, the strong associations observed between these rhizosphere families (e.g., *Bacillaceae, Rhizobiaceae*) and endophyte taxa such as Oxalobacteraceae and Lactobacillaceae may be mediated by oxalate metabolism. Oxalates, commonly exuded by plant roots or produced by soil microbes, can shape microbial communities by modifying pH and providing a carbon source, particularly stimulating oxalotrophic bacteria like Oxalobacteraceae ([Bibr bib0055][Bibr bib0040]). This suggests that oxalate-related interactions might contribute to functional connectivity between root and endophyte communities. Interestingly, only PER showed perfect negative correlations (ρ = –1.000), involving underexplored taxa from the rhizosphere (e.g., KD4–96, Caldilineaceae), and Promicromonosporaceae, Pseudomonadaceae, and Phycisphaeraceae from the endosphere. This divergence suggests that plant genotype plays a major role in shaping the structure and cross-interactions -of associated microbiota in endosphere and rhizosphere niches (Schweitzer et al., 2008) or niche competition or exclusion driven by microbial inoculation. Our findings highlighted that PER responded with broader and more functionally diverse microbial associations, potentially enhancing nutrient mobilization and plant protection, supporting previous findings on genotype-specific microbiome assembly and interaction strength (Wagner et al., 2016; Wei et al., 2019; Herrera Paredes et al., 2018). Overall, these networks confirm that microbial connectivity is not only niche-specific but also variety-dependent, with potential implications for targeted microbiome management and cultivar-specific inoculation strategies.

## 5 Conclusions

In conclusion, this study highlights for the first time the complexity and specificity of microbial community dynamics in sunflower rhizosphere and endosphere, shaped by plant genotype, microbial treatments and year-specific responses. Clear structural and compositional differences were observed between LST and PER varieties, with distinct taxonomic signatures and cross- interactions between endosphere and rhizosphere niches. Microbial inoculants significantly modulated community structure, particularly in the rhizosphere, and promoted the selective enrichment of beneficial taxa such as Pseudomonadaceae*,* Lactobacillaceae*,* Streptomycetaceae*,* Comamonadaceae, and Actinomycetaceae. Strong correlations between key microbial families across endosphere and rhizosphere habitats suggest functional complementarity and potential microbial transfer, which were often treatment dependent. These findings highlight the potential of targeted microbial management to enhance plant–microbe interactions and support the development of variety-specific microbiome engineering strategies for sustainable crop improvement.

## Ethics approval

The present work does not require an ethical approval.

## Source of biological material

Seeds were purchased or generously donated by seed-producing companies, whereas microorganisms used were isolated within the NO PROBleMS project (grant 777,760), and deposited at the University of Bologna microbial collection (BUSCOB).

## Statement on animal welfare

NA.

## Data availability

NGS raw sequence data have been submitted to the NCBI repository under the Sequence Read Archive (SRA) databases under the Bioproject N° PRJNA1311970, biosamples from SAMN50845502 to SAMN50845789 for rhizosphere and from SAMN50848321 to SAMN50848602 for endophytes.

## Funding

This study received the financial support of the project PRIN2022-IMPLICIT - Improving soil-plant-insect interactions to promote pollinators”, proposal code 2022NMAPEL - CUP J53D23006680006, Ministry of University and Research. This study was also carried out within the framework of Agritech National Research Center and received funding from the European Union Next-Generation EU (PIANO NAZIONALE DI RIPRESA E RESILIENZA (PNRR) – MISSIONE 4 COMPONENTE 2, INVESTIMENTO 1.4 – D.D. 1032 17/06/2022, CN00000022).

## Declaration of generative AI and AI-assisted technologies in the writing process

During the preparation of this work the author(s) used ChatGPT and Grammarly in order to improve the readability and language of the manuscript. After using this tool/service, the author(s) reviewed and edited the content as needed and take(s) full responsibility for the content of the published article.

## Declaration of competing interest

The authors declare that they have no known competing financial interests or personal relationships that could have appeared to influence the work reported in this paper.

## Appendix Supplementary materials

Image, application 1

Image, application 2

Image, application 3
