# Supplementary material for: PGPB administration influences the cross-talk and interactions of rhizosphere and endophyte microbial communities in sunflower
Source: Curr Res Microb Sci. 2025 Sep 25;9:100478. doi: 10.1016/j.crmicr.2025.100478 (PMC12554124; doi:10.1016/j.crmicr.2025.100478)
Supplement: Supplementary file 3 [file mmc3.pdf]

Supplementary Materials for:

PGPB administration influences the cross-talk and interactions of rhizosphere and endophyte microbial communities in sunflower

Chiara Braglia[1], Daniele Alberoni[1*], Loredana Baffoni[1], Sergio Angeli[2] and Diana Di Gioia[1]

[1]Dipartimento di Scienze e Tecnologie Agro-Alimentari, University of Bologna, Viale Fanin 44, 40127, Bologna, Italy;

[2]Faculty of Agricultural, Environmental and Food Sciences, Free University of Bozen-Bolzano, 39100 Bolzano, Italy

Chiara Braglia https://orcid.org/0000-0002-1637-896X

Daniele Alberoni: https://orcid.org/0000-0002-2394-2880

Loredana Baffoni: https://orcid.org/0000-0001-5313-5871

Sergio Angeli: https://orcid.org/0000-0002-8463-7476

Diana Di Gioia: https://orcid.org/0000-0002-0181-1572

[*]Corresponding Author: daniele.alberoni@unibo.it

Keywords: *Helianthus annuus*, Beneficial Bacteria, Microbial networks, Microbiome-plant genotipe interaction, Endophytic bacteria, Rhizosphere microbiota dynamics

Table S1. Primers used in this study.

Target	Primer Name	Sequence (5′-3′)	Amplicon size (bp)
16S rRNA V3-V4	Pro341-F	CCTACGGGNBGCASCAG	465
	Pro805-R	GACTACNVGGGTATCTAATCC	

α- and β-diversity considering both year (2023 and 2024)

Table S2. Pielou's evenness α-diversity index of the sunflower rhizosphere across the complete dataset (years 2023 and 2024). Statistical analysis was performed using Kruskal–Wallis test on the whole dataset and pairwise comparisons with FDR correction (Benjamini–Hochberg).

Comparison	Subsets	*p*-value	*p*-value corrected FDR-BH
1st year vs 2nd year of field tests	-	0.0009	
Year *vs* all experimental condition		0.0020	
	CTR 2023 *vs* all TRT 2023	0.0440	0.059
	CTR 2024 *vs* all TRT 2024	0.6700	> 0.1
	CTR 2023 *vs* CTR 2024	0.8700	> 0.1
	All TRT 2023 *vs* all TRT 2024	0.0003	0.00136
Year *vs* all experimental condition *vs* genotype		0.0009	> 0.1
	CTR LST 2023 *vs* all TRT LST 2023	0.0095	0.0571
	CTR PER 2023 *vs* all TRT PER 2023	0.9500	> 0.1
	CTR LST 2024 *vs* all TRT LST 2024	0.9900	> 0.1
	CTR PER 2024 *vs* all TRT PER 2024	0.5200	> 0.1
	CTR LST 2023 *vs* CTR LST 2024	0.0490	> 0.1
	CTR PER 2023 *vs* CTR PER 2024	0.1260	> 0.1
	All TRT LST 2023 *vs* all TRT PER 2023	0.1850	> 0.1
	All TRT LST 2024 *vs* all TRT PER 2024	0.7680	> 0.1
	CTR LST 2023 *vs* CTR PER 2023	0.0490	> 0.1
	CTR LST 2024 *vs* CTR PER 2024	0.8270	> 0.1
	All TRT LST 2023 *vs* all TRT LST 2024	0.0017	0.021
	All TRT PER 2023 *vs* all TRT PER 2024	0.0580	> 0.1
year *vs* bacterial genus *vs* genotype		0.0071	
	BAC LST 2023 *vs* BAC PER 2023	0.6240	> 0.1
	BAC LST 2023 *vs* CTR LST 2023	0.0139	> 0.1
	BAC LST 2023 *vs* LAC LST 2023	0.0153	> 0.1
	BAC LST 2023 *vs* PAE LST 2023	0.7120	> 0.1
	BAC LST 2023 *vs* BAC LST 2024	0.0728	> 0.1
	BAC PER 2023 *vs* CTR PER 2023	0.8230	> 0.1
	BAC PER 2023 *vs* LAC PER 2023	0.5710	> 0.1
	BAC PER 2023 *vs* PAE PER 2023	0.8688	> 0.1
	BAC PER 2023 *vs* BAC PER 2024	0.4478	> 0.1
	CTR LST 2023 *vs* CTR PER 2023	0.0495	> 0.1
	CTR LST 2023 *vs* LAC LST 2023	0.0179	> 0.1
	CTR LST 2023 *vs* PAE LST 2023	0.0388	> 0.1
	CTR LST 2023 *vs* CTR LST 2024	0.0495	> 0.1
	CTR PER 2023 *vs* LAC PER 2023	0.9263	> 0.1
	CTR PER 2023 *vs* PAE PER 2023	0.6547	> 0.1
	CTR PER 2023 *vs* CTR PER 2024	0.1266	> 0.1
	LAC LST 2023 *vs* PAE LST 2023	0.0170	> 0.1
	LAC LST 2023 *vs* LAC LST 2024	0.0063	> 0.1
	LAC PER 2023 *vs* PAE PER 2023	0.6407	> 0.1
	LAC PER 2023 *vs* LAC PER 2024	0.1451	> 0.1
	PAE LST 2023 *vs* PAE PER 2023	0.8551	> 0.1
	PAE LST 2023 *vs* PAE LST 2024	0.5218	> 0.1
	PAE LST 2023 *vs* PAE PER 2024	0.2012	> 0.1
	BAC LST 2024 *vs* BAC PER 2024	0.3113	> 0.1

	BAC LST 2024 *vs* CTR LST 2024	0.7630	> 0.1
	BAC LST 2024 *vs* LAC LST 2024	0.1463	> 0.1
	BAC LST 2024 *vs* PAE LST 2024	0.3503	> 0.1
	BAC PER 2024*vs* CTR PER 2024	0.6153	> 0.1
	BAC PER 2024 *vs* LAC PER 2024	0.9180	> 0.1
	BAC PER 2024 *vs* PAE PER 2024	0.1251	> 0.1
	CTR LST 2024 *vs* CTR PER 2024	0.8272	> 0.1
	CTR LST 2024 *vs* LAC LST 2024	0.4250	> 0.1
	CTR LST 2024 *vs* PAE LST 2024	1.0000	> 0.1
	CTR PER 2024 *vs* LAC PER 2024	0.9263	> 0.1
	CTR PER 2024 *vs* PAE PER 2024	0.1967	> 0.1
	LAC LST 2024 *vs* LAC PER 2024	0.5604	> 0.1
	LAC LST 2024 *vs* PAE LST 2024	0.8864	> 0.1
	LAC PER 2024 *vs* PAE PER 2024	0.1255	> 0.1
	PAE LST 2024 *vs* PAE PER 2024	0.0546	> 0.1

Table S3. Faith PD α-diversity index of the sunflower rhizosphere across the complete dataset (year 2023 and year 2024). Statistical analysis was performed using Kruskal–Wallis test on the whole dataset and pairwise comparisons with FDR correction (Benjamini–Hochberg).

Comparison	Subsets	*p*-value	*p*-value corrected FDR-BH
1st year vs 2nd year of field tests	-	0.0223	
Year *vs* all experimental condition		0.1490	
	CTR 2023 *vs* all TRT 2023	0.8626	> 0.1
	CTR 2024 *vs* all TRT 2024	0.8022	> 0.1
	CTR 2023 *vs* CTR 2024	0.3366	> 0.1
	All TRT 2023 *vs* all TRT 2024	0.0276	0.0737
Year *vs* all experimental condition *vs* genotype		0.3078	
	CTR LST 2023 *vs* all TRT LST 2023	0.6799	> 0.1
	CTR PER 2023 *vs* all TRT PER 2023	0.6163	> 0.1
	CTR LST 2024 *vs* all TRT LST 2024	0.4694	> 0.1
	CTR PER 2024 *vs* all TRT PER 2024	0.2462	> 0.1
	CTR LST 2023 *vs* CTR LST 2024	0.2752	> 0.1
	CTR PER 2023 *vs* CTR PER 2024	0.8272	> 0.1
	All TRT LST 2023 *vs* all TRT PER 2023	0.4725	> 0.1
	All TRT LST 2024 *vs* all TRT PER 2024	0.9344	> 0.1
	CTR LST 2023 *vs* CTR PER 2023	0.5127	> 0.1
	CTR LST 2024 *vs* CTR PER 2024	0.1266	> 0.1
	All TRT LST 2023 *vs* all TRT LST 2024	0.2040	> 0.1
	All TRT PER 2023 *vs* all TRT PER 2024	0.0518	> 0.1
Year *vs* bacterial genus *vs* genotype		0.5259	
	BAC LST 2023 *vs* BAC PER 2023	0.5464	> 0.1
	BAC LST 2023 *vs* CTR LST 2023	0.9109	> 0.1
	BAC LST 2023 *vs* LAC LST 2023	0.4605	> 0.1
	BAC LST 2023 *vs* PAE LST 2023	0.3763	> 0.1
	BAC LST 2023 *vs* BAC LST 2024	0.4076	> 0.1
	BAC PER 2023 *vs* CTR PER 2023	0.9109	> 0.1
	BAC PER 2023 *vs* LAC PER 2023	0.8208	> 0.1
	BAC PER 2023 *vs* PAE PER 2023	0.0575	> 0.1
	BAC PER 2023 *vs* BAC PER 2024	0.5575	> 0.1
	CTR LST 2023 *vs* CTR PER 2023	0.2127	> 0.1
	CTR LST 2023 *vs* LAC LST 2023	0.3980	> 0.1
	CTR LST 2023 *vs* PAE LST 2023	0.4385	> 0.1
	CTR LST 2023 *vs* CTR LST 2024	0.2752	> 0.1
	CTR PER 2023 *vs* LAC PER 2023	0.6439	> 0.1
	CTR PER 2023 *vs* PAE PER 2023	0.0526	> 0.1
	CTR PER 2023 *vs* CTR PER 2024	0.8272	> 0.1
	LAC LST 2023 *vs* LAC PER 2023	0.5676	> 0.1
	LAC LST 2023 *vs* PAE LST 2023	1.0000	> 0.1
	LAC LST 2023 *vs* LAC LST 2024	0.3797	> 0.1

	LAC PER 2023 *vs* PAE PER 2023	0.0196	> 0.1
	LAC PER 2023 *vs* LAC PER 2024	0.1711	> 0.1
	PAE LST 2023 *vs* PAE PER 2023	0.0176	> 0.1
	PAE LST 2023 *vs* PAE LST 2024	0.7487	> 0.1
	PAE LST 2023 *vs* PAE PER 2024	0.0284	> 0.1
	BAC LST 2024 *vs* BAC PER 2024	0.8743	> 0.1
	BAC LST 2024 *vs* CTR LST 2024	0.2689	> 0.1
	BAC LST 2024 *vs* LAC LST 2024	0.3968	> 0.1
	BAC LST 2024 *vs* PAE LST 2024	0.2861	> 0.1
	BAC PER 2024 *vs* CTR PER 2024	0.2689	> 0.1
	BAC PER 2024 *vs* LAC PER 2024	0.6807	> 0.1
	BAC PER 2024 *vs* PAE PER 2024	0.5485	> 0.1
	CTR LST 2024 *vs* CTR PER 2024	0.1266	> 0.1
	CTR LST 2024 *vs* LAC LST 2024	0.7324	> 0.1
	CTR LST 2024 *vs* PAE LST 2024	1.0000	> 0.1
	CTR PER 2024 *vs* LAC PER 2024	0.4053	> 0.1
	CTR PER 2024 *vs* PAE PER 2024	0.3017	> 0.1
	LAC LST 2024 *vs* LAC PER 2024	0.7110	> 0.1
	LAC LST 2024 *vs* PAE LST 2024	0.6682	> 0.1
	LAC PER 2024 *vs* PAE PER 2024	0.7236	> 0.1
	PAE LST 2024 *vs* PAE PER 2024	0.6309	> 0.1

Table S4. Unweighted unifrac index of the sunflower rhizosphere across the complete dataset (year 2023 and year 2024). Statistical analysis was performed using Kruskal–Wallis test on the whole dataset and pairwise comparisons with FDR correction (Benjamini–Hochberg).

**Comparison**	**Subsets**	***p*-value**	***p*-value corrected FDR-BH**
Year *vs* all experimental condition *vs* genotype			
	BAC LST 2023 *vs* BAC PER 2023	0.291	> 0.1
	BAC LST 2023 *vs* CTR LST 2023	0.201	> 0.1
	BAC LST 2023 *vs* BAC LST 2024	0.001	0.006
	BAC PER 2023 *vs* CTR PER 2023	0.643	> 0.1
	BAC PER 2023 *vs* BAC PER 2024	0.001	> 0.1
	CTR LST 2023 *vs* CTR PER 2023	0.777	> 0.1
	CTR LST 2023 *vs* CTR LST 2024	0.103	> 0.1
	CTR PER 2023 *vs* CTR PER 2024	0.116	> 0.1
	BAC LST 2024 *vs* BAC PER 2024	0.872	> 0.1
	BAC LST 2024 *vs* CTR LST 2024	0.634	> 0.1
	BAC PER 2024 *vs* CTR PER 2024	0.022	> 0.1
	CTR LST 2024 *vs* CTR PER 2024	0.086	> 0.1
Year *vs* bacterial genus *vs* genotype			
	BAC LST 2023 *vs* BAC PER 2023	0.638	> 0.1
	BAC LST 2023 *vs* CTR LST 2023	0.002	0.008
	BAC LST 2023 *vs* LAC LST 2023	0.001	0.0067
	BAC LST 2023 *vs* PAE LST 2023	0.204	> 0.1
	BAC LST 2023 *vs* BAC LST 2024	0.001	0.0067
	BAC PER 2023 *vs* CTR PER 2023	0.270	> 0.1
	BAC PER 2023 *vs* LAC PER 2023	0.197	> 0.1
	BAC PER 2023 *vs* PAE PER 2023	0.417	> 0.1
	BAC PER 2023 *vs* BAC PER 2024	0.001	0.0067
	CTR LST 2023 *vs* CTR PER 2023	0.202	> 0.1
	CTR LST 2023 *vs* LAC LST 2023	0.011	0.040
	CTR LST 2023 *vs* PAE LST 2023	0.022	0.0677
	CTR LST 2023 *vs* CTR LST 2024	0.096	> 0.1
	CTR PER 2023 *vs* LAC PER 2023	0.012	0.040
	CTR PER 2023 *vs* PAE PER 2023	0.092	> 0.1
	CTR PER 2023 *vs* CTR PER 2024	0.107	> 0.1
	LAC LST 2023 *vs* LAC PER 2023	0.002	0.008
	LAC LST 2023 *vs* PAE LST 2023	0.001	0.0067
	LAC LST 2023 *vs* LAC LST 2024	0.001	0.0067
	LAC PER 2023 *vs* PAE PER 2023	0.189	> 0.1

	LAC PER 2023 *vs* LAC PER 2024	0.001	0.0067
	PAE LST 2023 *vs* PAE PER 2023	0.086	> 0.1
	PAE LST 2023 *vs* PAE LST 2024	0.002	0.008
	PAE LST 2023 *vs* PAE PER 2024	0.002	0.008
	BAC LST 2024 *vs* BAC PER 2024	0.078	> 0.1
	BAC LST 2024 *vs* CTR LST 2024	0.227	> 0.1
	BAC LST 2024 *vs* LAC LST 2024	0.078	> 0.1
	BAC LST 2024 *vs* PAE LST 2024	0.189	> 0.1
	BAC PER 2024 *vs* CTR PER 2024	0.171	> 0.1
	BAC PER 2024 *vs* LAC PER 2024	0.608	> 0.1
	BAC PER 2024 *vs* PAE PER 2024	0.561	> 0.1
	CTR LST 2024 *vs* CTR PER 2024	0.897	> 0.1
	CTR LST 2024 *vs* LAC LST 2024	0.853	> 0.1
	CTR LST 2024 *vs* PAE LST 2024	0.716	> 0.1
	CTR PER 2024 *vs* LAC PER 2024	0.645	> 0.1
	CTR PER 2024 *vs* PAE PER 2024	0.096	> 0.1
	LAC LST 2024 *vs* LAC PER 2024	0.972	> 0.1
	LAC LST 2024 *vs* PAE LST 2024	0.317	> 0.1
	LAC PER 2024 *vs* PAE PER 2024	0.130	> 0.1
	PAE LST 2024 *vs* PAE PER 2024	0.179	> 0.1

Table S5. Bray-Curtis index of the sunflower rhizosphere across the complete dataset (year 2023 and year 2024). Statistical analysis was performed using Kruskal–Wallis test on the whole dataset and pairwise comparisons with FDR correction (Benjamini–Hochberg).

Comparison	Subsets	*p*-value	*p*-value corrected FDR-BH
Year *vs* all experimental condition *vs* genotype			
	BAC LST 2023 *vs* BAC PER 2023	0.195	> 0.1
	BAC LST 2023 *vs* CTR LST 2023	0.003	0.0120
	BAC LST 2023 *vs* BAC LST 2024	0.001	0.0060
	BAC PER 2023 *vs* CTR PER 2023	0.066	> 0.1
	BAC PER 2023 *vs* BAC PER 2024	0.001	0.0060
	CTR LST 2023 *vs* CTR PER 2023	0.185	> 0.1
	CTR LST 2023 *vs* CTR LST 2024	0.118	> 0.1
	CTR PER 2023 *vs* CTR PER 2024	0.083	> 0.1
	BAC LST 2024 *vs* BAC PER 2024	0.083	> 0.1
	BAC LST 2024 *vs* CTR LST 2024	0.617	> 0.1
	BAC PER 2024 *vs* CTR PER 2024	0.197	> 0.1
	CTR LST 2024 *vs* CTR PER 2024	0.900	> 0.1
Year *vs* bacterial genus *vs* genotype			
	BAC LST 2023 *vs* BAC PER 2023	0.638	> 0.1
	BAC LST 2023 *vs* CTR LST 2023	0.002	0.008
	BAC LST 2023 *vs* LAC LST 2023	0.001	0.006
	BAC LST 2023 *vs* PAE LST 2023	0.204	> 0.1
	BAC LST 2023 *vs* BAC LST 2024	0.001	0.006
	BAC PER 2023 *vs* CTR PER 2023	0.270	> 0.1
	BAC PER 2023 *vs* LAC PER 2023	0.197	> 0.1
	BAC PER 2023 *vs* PAE PER 2023	0.417	> 0.1
	BAC PER 2023 *vs* BAC PER 2024	0.001	0.006
	CTR LST 2023 *vs* CTR PER 2023	0.202	> 0.1
	CTR LST 2023 *vs* LAC LST 2023	0.011	0.040
	CTR LST 2023 *vs* PAE LST 2023	0.022	0.067
	CTR LST 2023 *vs* CTR LST 2024	0.096	> 0.1
	CTR PER 2023 *vs* LAC PER 2023	0.012	0.04
	CTR PER 2023 *vs* PAE PER 2023	0.092	> 0.1
	CTR PER 2023 *vs* CTR PER 2024	0.107	> 0.1
	LAC LST 2023 *vs* LAC PER 2023	0.002	0.008
	LAC LST 2023 *vs* PAE LST 2023	0.001	0.006
	LAC LST 2023 *vs* LAC LST 2024	0.001	0.006
	LAC PER 2023 *vs* PAE PER 2023	0.189	> 0.1
	LAC PER 2023 *vs* LAC PER 2024	0.001	0.006
	PAE LST 2023 *vs* PAE PER 2023	0.086	> 0.1

	PAE LST 2023 *vs* PAE LST 2024	0.002	0.008
	PAE LST 2023 *vs* PAE PER 2024	0.002	0.008
	BAC LST 2024 *vs* BAC PER 2024	0.078	> 0.1
	BAC LST 2024 *vs* CTR LST 2024	0.227	> 0.1
	BAC LST 2024 *vs* LAC LST 2024	0.078	> 0.1
	BAC LST 2024 *vs* PAE LST 2024	0.189	> 0.1
	BAC PER 2024 *vs* CTR PER 2024	0.171	> 0.1
	BAC PER 2024 *vs* LAC PER 2024	0.608	> 0.1
	BAC PER 2024 *vs* PAE PER 2024	0.561	> 0.1
	CTR LST 2024 *vs* CTR PER 2024	0.897	> 0.1
	CTR LST 2024 *vs* LAC LST 2024	0.853	> 0.1
	CTR LST 2024 *vs* PAE LST 2024	0.716	> 0.1
	CTR PER 2024 *vs* LAC PER 2024	0.645	> 0.1
	CTR PER 2024 *vs* PAE PER 2024	0.096	> 0.1
	LAC LST 2024 *vs* LAC PER 2024	0.972	> 0.1
	LAC LST 2024 *vs* PAE LST 2024	0.317	> 0.1
	LAC PER 2024 *vs* PAE PER 2024	0.130	> 0.1
	PAE LST 2024 *vs* PAE PER 2024	0.179	> 0.1

Table S6. Weighted unifrac index of the sunflower rhizosphere across the complete dataset (year 2023 and year 2024). Statistical analysis was performed using Kruskal–Wallis test on the whole dataset and pairwise comparisons with FDR correction (Benjamini–Hochberg).

**Comparison**	**Subsets**	***p*-value**	***p*-value corrected FDR-BH**
Year *vs* all experimental condition *vs* genotype		0.001	
	BAC LST 2023 *vs* BAC PER 2023	0.291	> 0.1
	BAC LST 2023 *vs* CTR LST 2023	0.201	> 0.1
	BAC LST 2023 *vs* BAC LST 2024	0.001	0.006
	BAC PER 2023 *vs* CTR PER 2023	0.643	> 0.1
	BAC PER 2023 *vs* BAC PER 2024	0.001	0.006
	CTR LST 2023 *vs* CTR PER 2023	0.777	> 0.1
	CTR LST 2023 *vs* CTR LST 2024	0.103	> 0.1
	CTR PER 2023 *vs* CTR PER 2024	0.116	> 0.1
	BAC LST 2024 *vs* BAC PER 2024	0.872	> 0.1
	BAC LST 2024 *vs* CTR LST 2024	0.634	> 0.1
	BAC PER 2024 *vs* CTR PER 2024	0.022	0.088
	CTR LST 2024 *vs* CTR PER 2024	0.086	> 0.1
Year *vs* bacterial genus *vs* genotype		0.001	
	BAC LST 2023 *vs* BAC PER 2023	0.321	> 0.1
	BAC LST 2023 *vs* CTR LST 2023	0.025	0.061
	BAC LST 2023 *vs* LAC LST 2023	0.001	0.006
	BAC LST 2023 *vs* PAE LST 2023	0.476	> 0.1
	BAC LST 2023 *vs* BAC LST 2024	0.001	0.006
	BAC PER 2023 *vs* CTR PER 2023	0.434	> 0.1
	BAC PER 2023 *vs* LAC PER 2023	0.344	> 0.1
	BAC PER 2023 *vs* PAE PER 2023	0.481	> 0.1
	BAC PER 2023 *vs* BAC PER 2024	0.001	0.006
	CTR LST 2023 *vs* CTR PER 2023	0.103	> 0.1
	CTR LST 2023 *vs* LAC LST 2023	0.006	0.019
	CTR LST 2023 *vs* PAE LST 2023	0.021	0.056
	CTR LST 2023 *vs* CTR LST 2024	0.078	> 0.1
	CTR PER 2023 *vs* LAC PER 2023	0.137	> 0.1
	CTR PER 2023 *vs* PAE PER 2023	0.238	> 0.1
	CTR PER 2023 *vs* CTR PER 2024	0.101	> 0.1
	LAC LST 2023 *vs* LAC PER 2023	0.002	0.008
	LAC LST 2023 *vs* PAE LST 2023	0.001	0.006
	LAC LST 2023 *vs* LAC LST 2024	0.306	> 0.1
	LAC PER 2023 *vs* PAE PER 2023	0.001	0.006
	LAC PER 2023 *vs* LAC PER 2024	0.002	0.008
	PAE LST 2023 *vs* PAE PER 2023	0.002	0.008
	PAE LST 2023 *vs* PAE LST 2024	0.278	> 0.1

	PAE LST 2023 *vs* PAE PER 2024	0.015	0.043
	BAC LST 2024 *vs* BAC PER 2024	0.048	> 0.1
	BAC LST 2024 *vs* CTR LST 2024	0.324	> 0.1
	BAC LST 2024 *vs* LAC LST 2024	0.524	> 0.1
	BAC LST 2024 *vs* PAE LST 2024	0.148	> 0.1
	BAC PER 2024 *vs* CTR PER 2024	0.753	> 0.1
	BAC PER 2024 *vs* LAC PER 2024	0.237	> 0.1
	BAC PER 2024 *vs* PAE PER 2024	0.55	> 0.1
	CTR LST 2024 *vs* CTR PER 2024	0.004	0.014
	CTR LST 2024 *vs* LAC LST 2024	0.321	> 0.1
	CTR LST 2024 *vs* PAE LST 2024	0.025	0.061
	CTR PER 2024 *vs* LAC PER 2024	0.001	0.0064
	CTR PER 2024 *vs* PAE PER 2024	0.476	> 0.1
	LAC LST 2024 *vs* LAC PER 2024	0.001	0.006
	LAC LST 2024 *vs* PAE LST 2024	0.434	> 0.1
	LAC PER 2024 *vs* PAE PER 2024	0.344	> 0.1
	PAE LST 2024 *vs* PAE PER 2024	0.481	> 0.1

α- and β-diversity considering the year 2023

Table S7. Pielou evenness α-diversity index of the sunflower rhizosphere on the year 2023. Statistical analysis was performed using Kruskal–Wallis test on the whole dataset and pairwise comparisons with FDR correction (Benjamini–Hochberg).

Comparison	Subsets	*p*-value	*p*-value corrected FDR-BH
all experimental conditions *vs* genotype		0.0009	> 0.1
	All TRT LST 2023 *vs* all TRT PER 2023	0.1666	0.2221
	CTR LST 2023 *vs* all TRT LST 2023	0.0139	0.0556
	CTR PER 2023 *vs* all TRT PER 2023	0.7711	0.7711
	CTR LST 2023 *vs* CTR PER 2023	0.0495	0.0991
bacterial genus *vs* genotype		0.0071	
	BAC LST 2023 *vs* BAC PER 2023	0.8433	> 0.1
	BAC LST 2023 *vs* CTR LST 2023	0.0253	0.081
	BAC LST 2023 *vs* LAC LST 2023	0.0015	0.017
	BAC LST 2023 *vs* PAE LST 2023	0.9412	> 0.1
	BAC PER 2023 *vs* CTR PER 2023	0.5940	> 0.1
	BAC PER 2023 *vs* LAC PER 2023	0.6550	> 0.1
	BAC PER 2023 *vs* PAE PER 2023	0.9652	> 0.1
	CTR LST 2023 *vs* CTR PER 2023	0.0495	> 0.1
	CTR LST 2023 *vs* LAC LST 2023	0.0125	0.050
	CTR LST 2023 *vs* PAE LST 2023	0.0710	> 0.1
	CTR PER 2023 *vs* LAC PER 2023	0.7815	> 0.1
	CTR PER 2023 *vs* PAE PER 2023	0.6550	> 0.1
	LAC LST 2023 *vs* LAC PER 2023	0.0071	0.037
	LAC LST 2023 *vs* PAE LST 2023	0.0022	0.017
	LAC PER 2023 *vs* PAE PER 2023	0.6410	> 0.1
	PAE LST 2023 *vs* PAE PER 2023	1.0000	1.0

Table S8. Faith PD α-diversity index of the sunflower rhizosphere on the year 2023. Statistical analysis was performed using Kruskal–Wallis test on the whole dataset and pairwise comparisons with FDR correction (Benjamini–Hochberg).

Comparison	Subsets	*p*-value	*p*-value corrected FDR-BH
all experimental conditions *vs* genotype		0.79	
	All TRT LST 2023 vs all TRT PER 2023	0.938	> 0.1
	CTR LST 2023 vs all TRT LST 2023	0.155	> 0.1
	CTR PER 2023 vs all TRT PER 2023	0.176	> 0.1
	CTR LST 2023 vs CTR PER 2023	0.047	> 0.1
bacterial genus *vs* genotype		0.420	
	BAC LST 2023 vs BAC PER 2023	0.843	> 0.1
	BAC LST 2023 vs CTR LST 2023	0.910	> 0.1
	BAC LST 2023 vs LAC LST 2023	0.282	> 0.1
	BAC LST 2023 vs PAE LST 2023	0.268	> 0.1
	BAC PER 2023 vs CTR PER 2023	0.952	> 0.1
	BAC PER 2023 vs LAC PER 2023	0.456	> 0.1
	BAC PER 2023 vs PAE PER 2023	0.149	> 0.1
	CTR LST 2023 vs CTR PER 2023	0.827	> 0.1
	CTR LST 2023 vs LAC LST 2023	0.405	> 0.1
	CTR LST 2023 vs PAE LST 2023	0.438	> 0.1
	CTR PER 2023 vs LAC PER 2023	0.781	> 0.1
	CTR PER 2023 vs PAE PER 2023	0.179	> 0.1
	LAC LST 2023 *vs* LAC PER 2023	0.507	> 0.1
	LAC LST 2023 *vs* PAE LST 2023	0.723	> 0.1
	LAC PER 2023 *vs* PAE PER 2023	0.038	> 0.1
	PAE LST 2023 *vs* PAE PER 2023	0.028	> 0.1
bacterial strain treatment*			
	DAN39 *vs* SP12	0.010	0.057

	DAN39 *vs* SP9	0.010	0.057
	DAN91 *vs* SP12	0.003	0.057
	DAN91 *vs* SP27	0.003	0.057
	DAN91 *vs* SP9	0.010	0.057
	K84 *vs* SP12	0.006	0.057
	K85 *vs* SP12	0.010	0.057
	LB9 *vs* SP12	0.010	0.057
	LB9 *vs* SP9	0.010	0.057
	SP12 *vs* SP43	0.003	0.057
	SP27 *vs* SP43	0.010	0.057
	SP43 *vs* SP9	0.010	0.057

* Here we show only pairwise comparisons with p < 0.1

Table S9. Unweighted unifrac index of the sunflower rhizosphere on the year 2023. Statistical analysis was performed using Kruskal–Wallis test on the whole dataset and pairwise comparisons with FDR correction (Benjamini–Hochberg).

Comparison	Subsets	*p*-value	*p*-value corrected FDR-BH
all experimental conditions *vs* genotype		0.545	
	BAC LST 2023 *vs* BAC PER 2023	0.422	> 0.1
	BAC LST 2023 *vs* CTR LST 2023	0.322	> 0.1
	BAC PER 2023 *vs* CTR PER 2023	0.625	> 0.1
	CTR LST 2023 *vs* CTR PER 2023	0.583	> 0.1
year *vs* bacterial genus *vs* genotype		0.035	
	BAC LST 2023 *vs* BAC PER 2023	0.642	> 0.1
	BAC LST 2023 *vs* CTR LST 2023	0.509	> 0.1
	BAC LST 2023 *vs* LAC LST 2023	0.009	0.058
	BAC LST 2023 *vs* PAE LST 2023	0.233	> 0.1
	BAC PER 2023 *vs* CTR PER 2023	0.879	> 0.1
	BAC PER 2023 *vs* LAC PER 2023	0.160	> 0.1
	BAC PER 2023 *vs* PAE PER 2023	0.600	> 0.1
	CTR LST 2023 *vs* CTR PER 2023	0.567	> 0.1
	CTR LST 2023 *vs* LAC LST 2023	0.074	> 0.1
	CTR LST 2023 *vs* PAE LST 2023	0.203	> 0.1
	CTR PER 2023 *vs* LAC PER 2023	0.136	> 0.1
	CTR PER 2023 *vs* PAE PER 2023	0.004	> 0.1
	LAC LST 2023 *vs* LAC PER 2023	0.004	0.052
	LAC LST 2023 *vs* PAE LST 2023	0.016	0.069
	LAC PER 2023 *vs* PAE PER 2023	0.509	> 0.1
	PAE LST 2023 *vs* PAE PER 2023	0.009	> 0.1

Table S10. Weighted unifrac index of the sunflower rhizosphere on the year 2023. Statistical analysis was performed using Kruskal–Wallis test on the whole dataset and pairwise comparisons with FDR correction (Benjamini–Hochberg).

Comparison	Subsets	*p*-value	*p*-value corrected FDR-BH
all experimental conditions *vs* genotype		0.015	
	BAC LST 2023 *vs* BAC PER 2023	0.191	> 0.1
	BAC LST 2023 *vs* CTR LST 2023	0.009	0.020
	BAC PER 2023 *vs* CTR PER 2023	0.010	0.020
	CTR LST 2023 *vs* CTR PER 2023	0.085	> 0.1
year *vs* bacterial genus *vs* genotype		0.001	
	BAC LST 2023 *vs* BAC PER 2023	0.001	0.016
	BAC LST 2023 *vs* CTR LST 2023	0.003	0.016
	BAC LST 2023 *vs* LAC LST 2023	0.004	0.016
	BAC LST 2023 *vs* PAE LST 2023	0.004	0.016
	BAC PER 2023 *vs* CTR PER 2023	0.027	0.085333
	BAC PER 2023 *vs* LAC PER 2023	0.032	0.085333

	BAC PER 2023 *vs* PAE PER 2023	0.095	0.217143
	CTR LST 2023 *vs* CTR PER 2023	0.11	0.22
	CTR LST 2023 *vs* LAC LST 2023	0.253	0.406667
	CTR LST 2023 *vs* PAE LST 2023	0.302	0.406667
	CTR PER 2023 *vs* LAC PER 2023	0.305	0.406667
	CTR PER 2023 *vs* PAE PER 2023	0.305	0.406667
	LAC LST 2023 *vs* LAC PER 2023	0.486	0.559
	LAC LST 2023 *vs* PAE LST 2023	0.518	0.559
	LAC PER 2023 *vs* PAE PER 2023	0.526	0.559
	PAE LST 2023 *vs* PAE PER 2023	0.559	0.559

Table S11. Bray-Curtis index of the sunflower rhizosphere on the year 2023. Statistical analysis was performed using Kruskal–Wallis test on the whole dataset and pairwise comparisons with FDR correction (Benjamini–Hochberg).

Comparison	Subsets	*p*-value	*p*-value corrected FDR-BH
all experimental conditions *vs* genotype		0.001	
	BAC LST 2023 *vs* BAC PER 2023	0.143	> 0.1
	BAC LST 2023 *vs* CTR LST 2023	0.002	0.008
	BAC PER 2023 *vs* CTR PER 2023	0.065	0.087
	CTR LST 2023 *vs* CTR PER 2023	0.199	> 0.1
year *vs* bacterial genus *vs* genotype		0.015	
	BAC LST 2023 *vs* BAC PER 2023	0.563	> 0.1
	BAC LST 2023 *vs* CTR LST 2023	0.007	0.022
	BAC LST 2023 *vs* LAC LST 2023	0.001	0.005
	BAC LST 2023 *vs* PAE LST 2023	0.22	> 0.1
	BAC PER 2023 *vs* CTR PER 2023	0.301	> 0.1
	BAC PER 2023 *vs* LAC PER 2023	0.208	> 0.1
	BAC PER 2023 *vs* PAE PER 2023	0.518	> 0.1
	CTR LST 2023 *vs* CTR PER 2023	0.197	> 0.1
	CTR LST 2023 *vs* LAC LST 2023	0.005	0.02
	CTR LST 2023 *vs* PAE LST 2023	0.029	> 0.1
	CTR PER 2023 *vs* LAC PER 2023	0.015	0.04
	CTR PER 2023 *vs* PAE PER 2023	0.077	> 0.1
	LAC LST 2023 *vs* LAC PER 2023	0.001	0.005
	LAC LST 2023 *vs* PAE LST 2023	0.001	0.005
	LAC PER 2023 *vs* PAE PER 2023	0.212	> 0.1
	PAE LST 2023 *vs* PAE PER 2023	0.095	> 0.1

α- and β-diversity considering the year 2024

Table S12. Pielou evenness α-diversity index of the sunflower rhizosphere on the year 2024. Statistical analysis was performed using Kruskal–Wallis test on the whole dataset and pairwise comparisons with FDR correction (Benjamini–Hochberg).

Comparison	Subsets	*p*-value	*p*-value corrected FDR-BH
all experimental conditions *vs* genotype		0.6565	
	All TRT LST 2024 *vs* all TRT PER 2024	0.2643	> 0.1
	CTR LST 2024 *vs* all TRT LST 2024	0.9557	> 0.1
	CTR PER 2024 *vs* all TRT PER 2024	0.4606	> 0.1
	CTR LST 2024 *vs* CTR PER 2024	0.8272	> 0.1
bacterial genus *vs* genotype		0.7292	
	BAC LST 2024 *vs* BAC PER 2024	0.1137	> 0.1
	BAC LST 2024 *vs* CTR LST 2024	0.9199	> 0.1

	BAC LST 2024 *vs* LAC LST 2024	0.3639	> 0.1
	BAC LST 2024 *vs* PAE LST 2024	0.5049	> 0.1
	BAC PER 2024 *vs* CTR PER 2024	0.3149	> 0.1
	BAC PER 2024 *vs* LAC PER 2024	0.1811	> 0.1
	BAC PER 2024 *vs* PAE PER 2024	0.5938	> 0.1
	CTR LST 2024 *vs* CTR PER 2024	0.8272	> 0.1
	CTR LST 2024 *vs* LAC LST 2024	0.3050	> 0.1
	CTR LST 2024 *vs* PAE LST 2024	1	> 0.1
	CTR PER 2024 *vs* LAC PER 2024	0.9263	> 0.1
	CTR PER 2024 *vs* PAE PER 2024	0.6056	> 0.1
	LAC LST 2024 *vs* LAC PER 2024	0.3683	> 0.1
	LAC LST 2024 *vs* PAE LST 2024	0.8864	> 0.1
	LAC PER 2024 *vs* PAE PER 2024	0.5556	> 0.1
	PAE LST 2024 *vs* PAE PER 2024	0.7488	> 0.1

Table S13. Faith PD α-diversity index of the sunflower rhizosphere on the year 2024. Statistical analysis was performed using Kruskal–Wallis test on the whole dataset and pairwise comparisons with FDR correction (Benjamini–Hochberg).

Comparison	Subsets	*p*-value	*p*-value corrected FDR-BH
all experimental conditions *vs* genotype		0.9847	
	All TRT LST 2024 vs all TRT PER 2024	0.8972	> 0.1
	CTR LST 2024 vs all TRT LST 2024	0.9114	> 0.1
	CTR PER 2024 vs all TRT PER 2024	1	> 0.1
	CTR LST 2024 vs CTR PER 2024	0.5127	> 0.1
bacterial genus *vs* genotype		0.9918	
	BAC LST 2024 vs BAC PER 2024	0.8494	> 0.1
	BAC LST 2024 vs CTR LST 2024	0.7630	> 0.1
	BAC LST 2024 vs LAC LST 2024	0.8087	> 0.1
	BAC LST 2024 vs PAE LST 2024	0.6407	> 0.1
	BAC PER 2024 vs CTR PER 2024	0.9199	> 0.1
	BAC PER 2024 vs LAC PER 2024	0.5371	> 0.1
	BAC PER 2024 vs PAE PER 2024	0.8415	> 0.1
	CTR LST 2024 vs CTR PER 2024	0.5127	> 0.1
	CTR LST 2024 vs LAC LST 2024	0.7324	> 0.1
	CTR LST 2024 vs PAE LST 2024	0.6056	> 0.1
	CTR PER 2024 vs LAC PER 2024	0.9263	> 0.1
	CTR PER 2024 vs PAE PER 2024	0.6056	> 0.1
	LAC LST 2024 *vs* LAC PER 2024	0.4273	> 0.1
	LAC LST 2024 *vs* PAE LST 2024	0.6682	> 0.1
	LAC PER 2024 *vs* PAE PER 2024	0.9062	> 0.1
	PAE LST 2024 *vs* PAE PER 2024	0.7488	> 0.1

Table S14. Unweighted unifrac index of the sunflower rhizosphere on the year 2024. Statistical analysis was performed using Kruskal–Wallis test on the whole dataset and pairwise comparisons with FDR correction (Benjamini–Hochberg).

Comparison	Subsets	*p*-value	*p*-value corrected FDR-BH
all experimental conditions *vs* genotype		0.011	
	BAC LST 2024 *vs* BAC PER 2024	0.0290	0.0580
	BAC LST 2024 *vs* CTR LST 2024	0.2760	> 0.1
	BAC PER 2024 *vs* CTR PER 2024	0.0150	0.0580
	CTR LST 2024 *vs* CTR PER 2024	0.1100	> 0.1
year *vs* bacterial genus *vs* genotype		0.005	
	BAC LST 2024 *vs* BAC PER 2024	0.0140	0.0747
	BAC LST 2024 *vs* CTR LST 2024	0.1040	> 0.1
	BAC LST 2024 *vs* LAC LST 2024	0.0030	0.0480
	BAC LST 2024 *vs* PAE LST 2024	0.7500	> 0.1
	BAC PER 2024 *vs* CTR PER 2024	0.0120	0.0747

	BAC PER 2024 *vs* LAC PER 2024	0.2270	> 0.1
	BAC PER 2024 *vs* PAE PER 2024	0.8710	> 0.1
	CTR LST 2024 *vs* CTR PER 2024	0.1080	> 0.1
	CTR LST 2024 *vs* LAC LST 2024	0.6750	> 0.1
	CTR LST 2024 *vs* PAE LST 2024	0.2460	> 0.1
	CTR PER 2024 *vs* LAC PER 2024	0.0390	> 0.1
	CTR PER 2024 *vs* PAE PER 2024	0.0440	> 0.1
	LAC LST 2024 *vs* LAC PER 2024	0.7690	> 0.1
	LAC LST 2024 *vs* PAE LST 2024	0.0530	> 0.1
	LAC PER 2024 *vs* PAE PER 2024	0.7280	> 0.1
	PAE LST 2024 *vs* PAE PER 2024	0.2090	> 0.1

Table S15. Weighted unifrac index of the sunflower rhizosphere on the year 2024. Statistical analysis was performed using Kruskal–Wallis test on the whole dataset and pairwise comparisons with FDR correction (Benjamini–Hochberg).

Comparison	Subsets	*p*-value	*p*-value corrected FDR-BH
all experimental conditions *vs* genotype		0.034	
	BAC LST 2024 *vs* BAC PER 2024	0.0110	0.0440
	BAC LST 2024 *vs* CTR LST 2024	0.6520	> 0.1
	BAC PER 2024 *vs* CTR PER 2024	0.3250	> 0.1
	CTR LST 2024 *vs* CTR PER 2024	0.4880	> 0.1
year *vs* bacterial genus *vs* genotype		0.067	
	BAC LST 2024 *vs* BAC PER 2024	0.0100	> 0.1
	BAC LST 2024 *vs* CTR LST 2024	0.2570	> 0.1
	BAC LST 2024 *vs* LAC LST 2024	0.0230	> 0.1
	BAC LST 2024 *vs* PAE LST 2024	0.6360	> 0.1
	BAC PER 2024 *vs* CTR PER 2024	0.4370	> 0.1
	BAC PER 2024 *vs* LAC PER 2024	0.7020	> 0.1
	BAC PER 2024 *vs* PAE PER 2024	0.6610	> 0.1
	CTR LST 2024 *vs* CTR PER 2024	0.5050	> 0.1
	CTR LST 2024 *vs* LAC LST 2024	0.8650	> 0.1
	CTR LST 2024 *vs* PAE LST 2024	0.3030	> 0.1
	CTR PER 2024 *vs* LAC PER 2024	0.6250	> 0.1
	CTR PER 2024 *vs* PAE PER 2024	0.2130	> 0.1
	LAC LST 2024 *vs* LAC PER 2024	0.7180	> 0.1
	LAC LST 2024 *vs* PAE LST 2024	0.0970	> 0.1
	LAC PER 2024 *vs* PAE PER 2024	0.5750	> 0.1
	PAE LST 2024 *vs* PAE PER 2024	0.0720	> 0.1

Table S16. Bray-Curtis index of the sunflower rhizosphere on the year 2024. Statistical analysis was performed using Kruskal–Wallis test on the whole dataset and pairwise comparisons with FDR correction (Benjamini–Hochberg).

Comparison	Subsets	*p*-value	*p*-value corrected FDR-BH
all experimental conditions *vs* genotype		0.131	
	BAC LST 2024 *vs* BAC PER 2024	0.0620	> 0.1
	BAC LST 2024 *vs* CTR LST 2024	0.5950	> 0.1
	BAC PER 2024 *vs* CTR PER 2024	0.0700	> 0.1
	CTR LST 2024 *vs* CTR PER 2024	0.5600	> 0.1
year *vs* bacterial genus *vs* genotype		0.063	
	BAC LST 2024 *vs* BAC PER 2024	0.0420	> 0.1
	BAC LST 2024 *vs* CTR LST 2024	0.2370	> 0.1
	BAC LST 2024 *vs* LAC LST 2024	0.0280	> 0.1
	BAC LST 2024 *vs* PAE LST 2024	0.2280	> 0.1
	BAC PER 2024 *vs* CTR PER 2024	0.2280	> 0.1
	BAC PER 2024 *vs* LAC PER 2024	0.4220	> 0.1

	BAC PER 2024 *vs* PAE PER 2024	0.5440	> 0.1
	CTR LST 2024 *vs* CTR PER 2024	0.7820	> 0.1
	CTR LST 2024 *vs* LAC LST 2024	0.7890	> 0.1
	CTR LST 2024 *vs* PAE LST 2024	0.8470	> 0.1
	CTR PER 2024 *vs* LAC PER 2024	0.6100	> 0.1
	CTR PER 2024 *vs* PAE PER 2024	0.0450	> 0.1
	LAC LST 2024 *vs* LAC PER 2024	0.7600	> 0.1
	LAC LST 2024 *vs* PAE LST 2024	0.3300	> 0.1
	LAC PER 2024 *vs* PAE PER 2024	0.0390	> 0.1
	PAE LST 2024 *vs* PAE PER 2024	0.1380	> 0.1

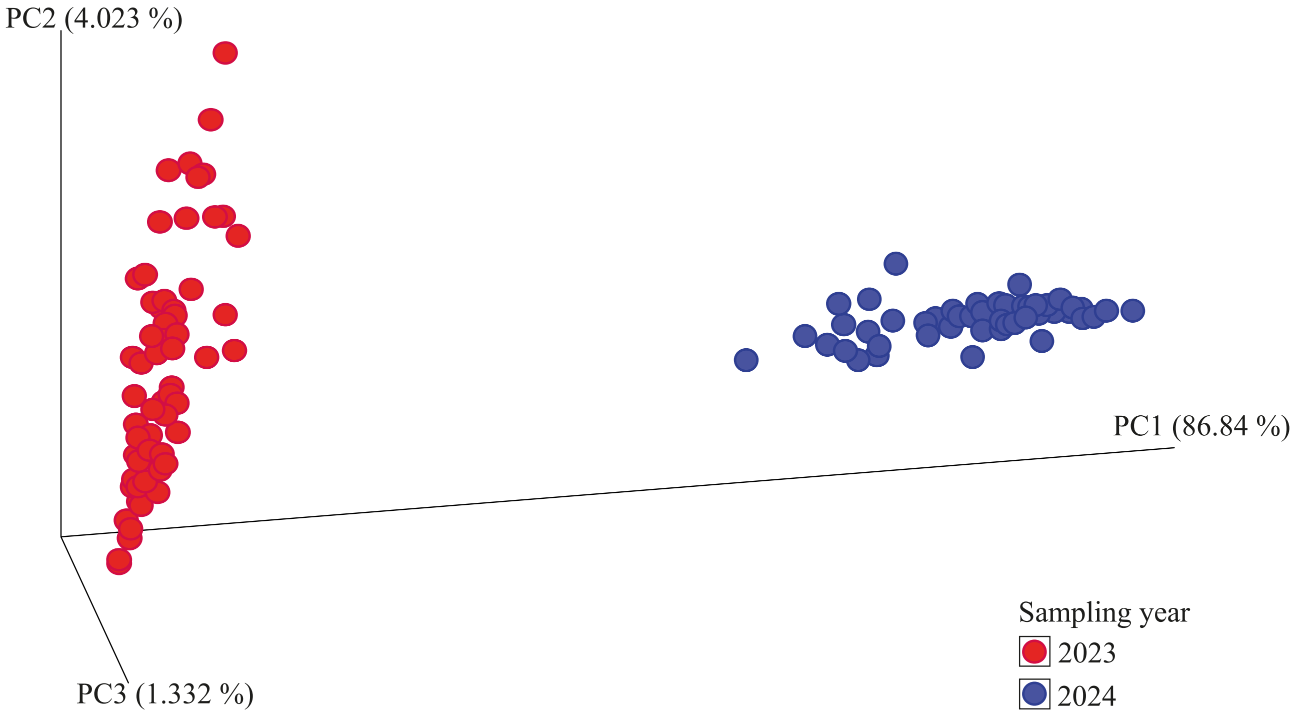

Figure S1: Principal Coordinates Analysis (PCoA) based on Bray–Curtis distances showing microbial community structure across sampling years (2023 and 2024).

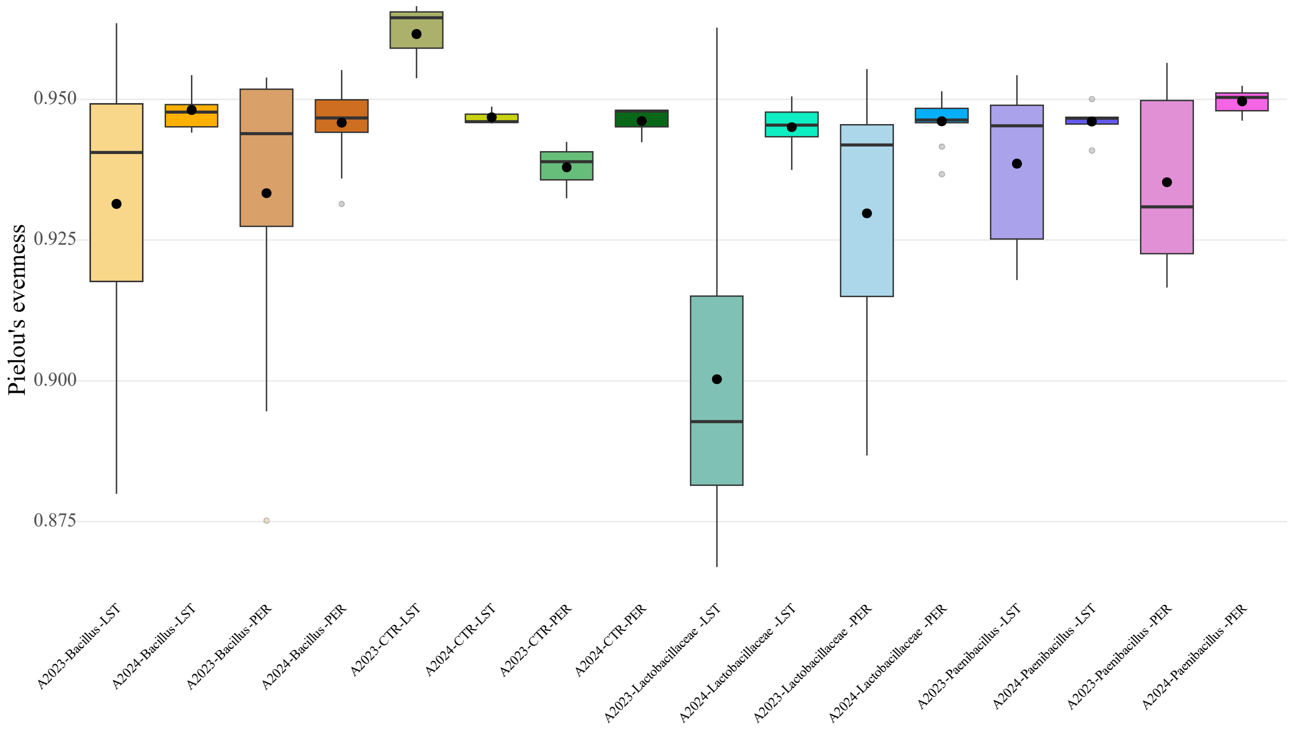

Figure S2: Boxplots illustrating Pielou’s Evenness index in the rhizosphere microbiome (datasets from 2023 and 2024). Statistical tests and pairwise significant comparisons (year × bacterial genus × genotype) are reported in Table S2. Boxplots indicate variability within treatments, and black dots represent mean values.

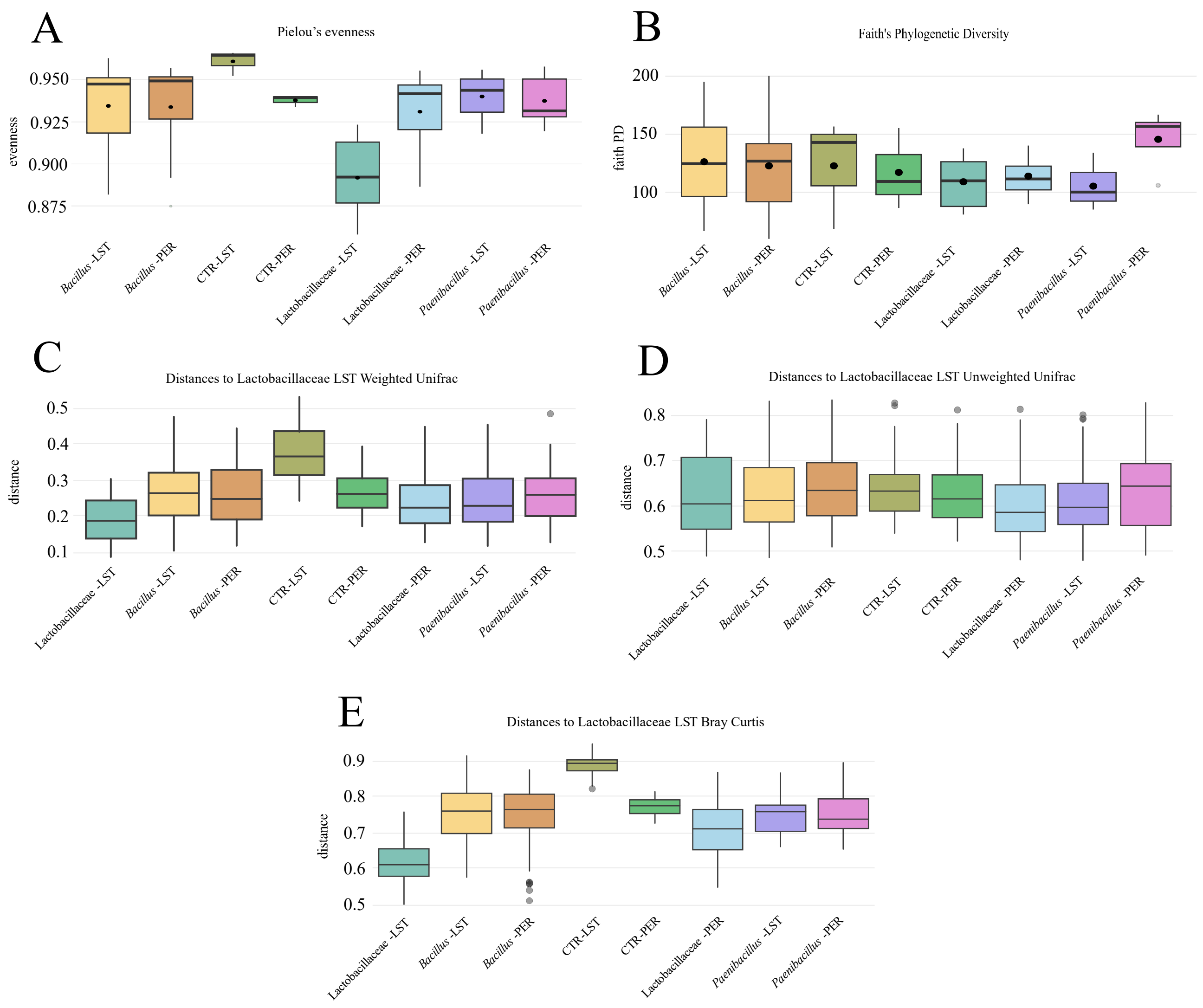

Figure S3A – S3E: Boxplots showing diversity metrics of the rhizosphere microbiome (2023 dataset). (A) Pielou's evenness index, see Table S7. (B) Faith's phylogenetic diversity, Table S8. (C) Distances to Lactobacillaceae LST based on weighted UniFrac, Table S10. (D) Distances to Lactobacillaceae LST based on unweighted UniFrac, Table S9. (E) Distances to Lactobacillaceae LST based on Bray–Curtis dissimilarity, Table S11.

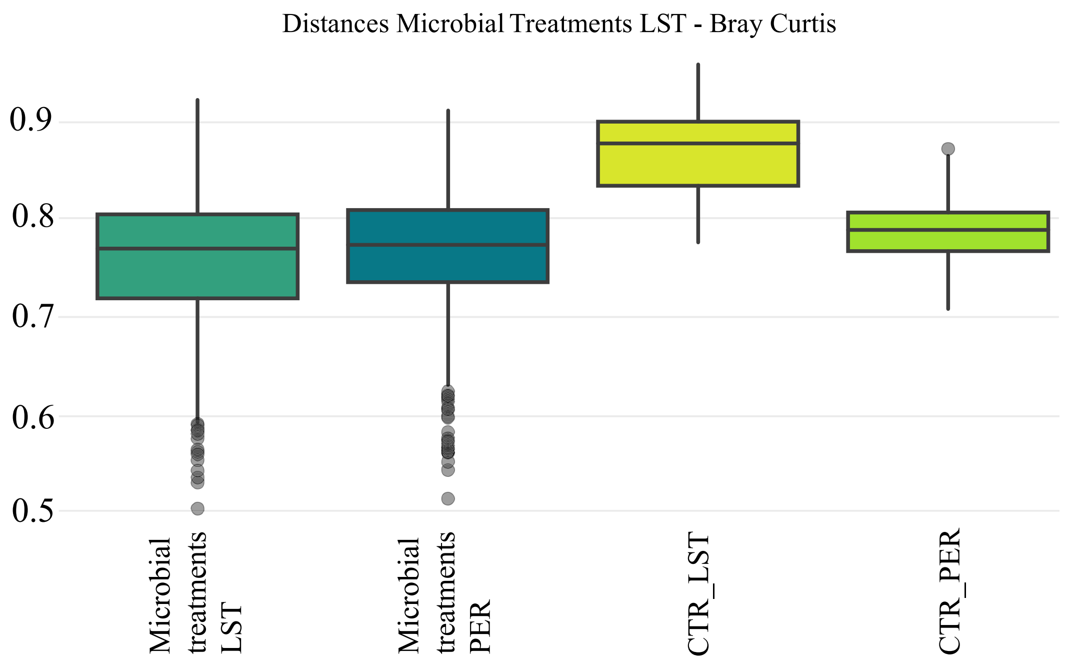

Figure S4: Boxplots illustrating Bray–Curtis distances to MicrobialTreatments_LST (2023 dataset). Statistical tests and pairwise significant comparisons (treatment × genotype) are reported in Table S11.

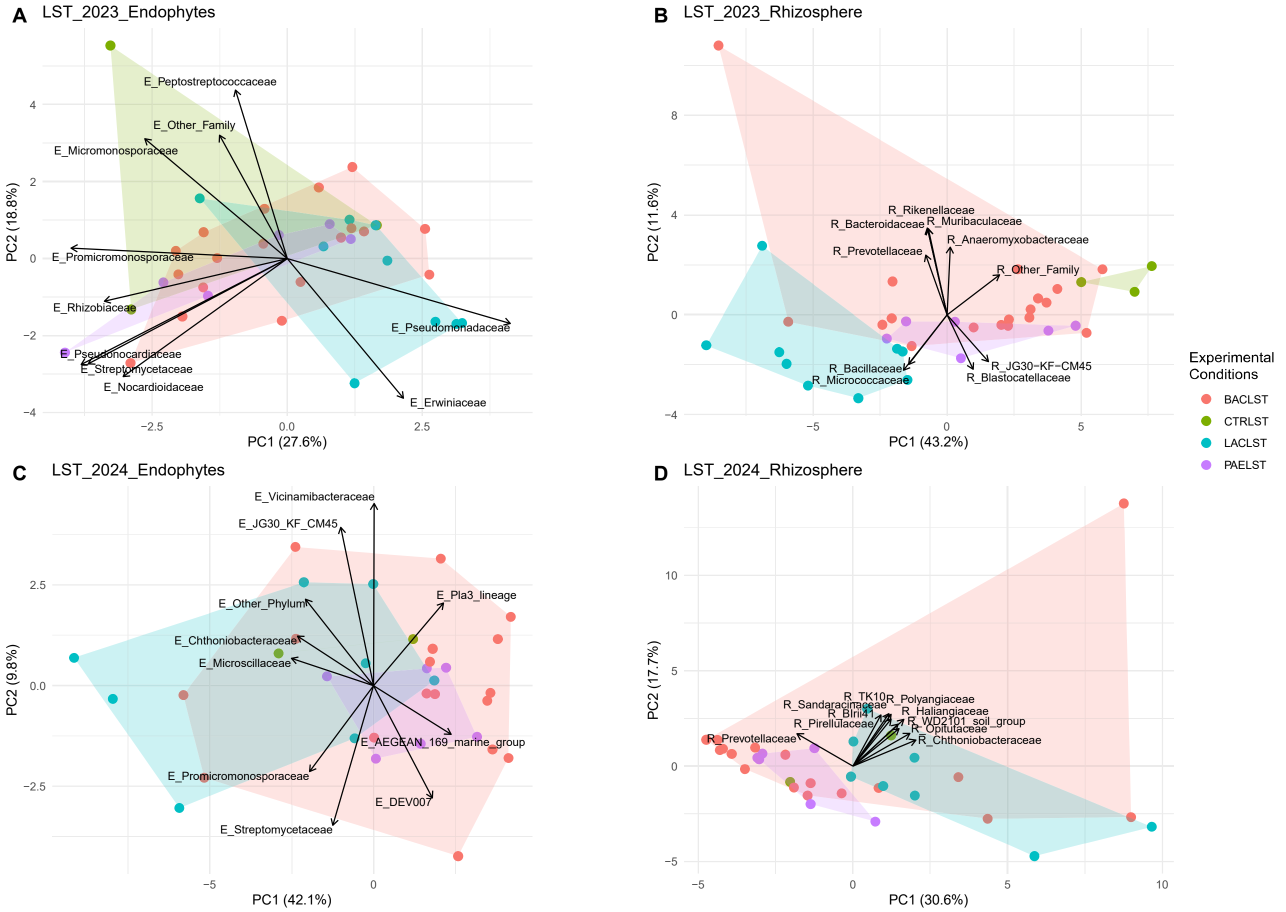

Figure S5: Principal component analysis score plot on the effect of the different microbial consortia on the LST variety (*H. annuus)* rhizosphere (R_) and endophytes (E_) microbial community detected by NGS analysis in the experiment performed in 2023 and 2024. [CTR] control; [BAC] *Bacillus*; [LAC] *Lactobacillus*; [PAE] *Paenibacillus.*

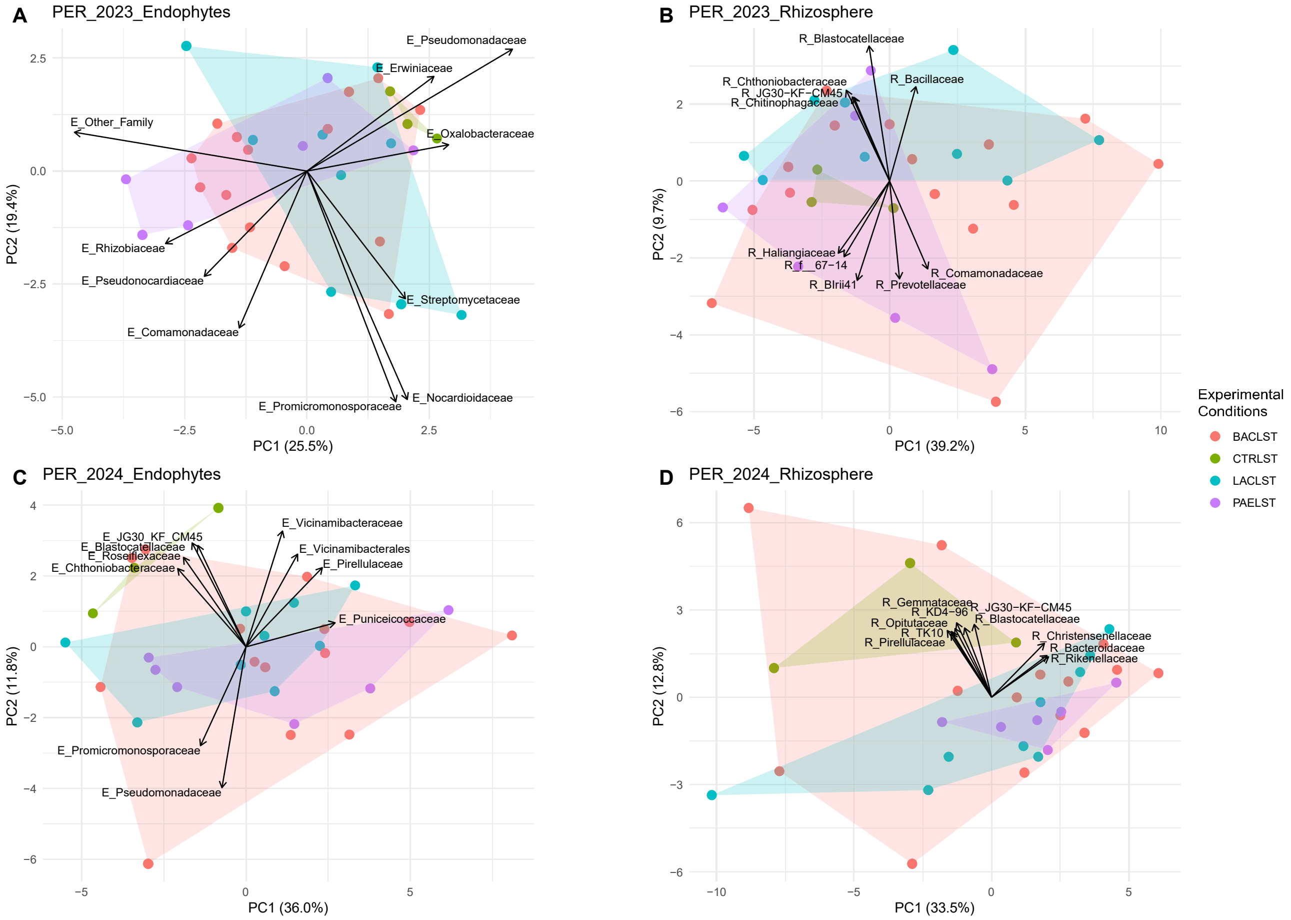

Figure S6: Principal component analysis score plot on the effect of the different microbial consortia on the PER variety (*H. annuus)* rhizosphere (R_) and endophytes (E_) microbial community detected by NGS analysis in the experiment performed in 2023 and 2024. [CTR] control; [BAC] *Bacillus*; [LAC] *Lactobacillus*; [PAE] *Paenibacillus.*

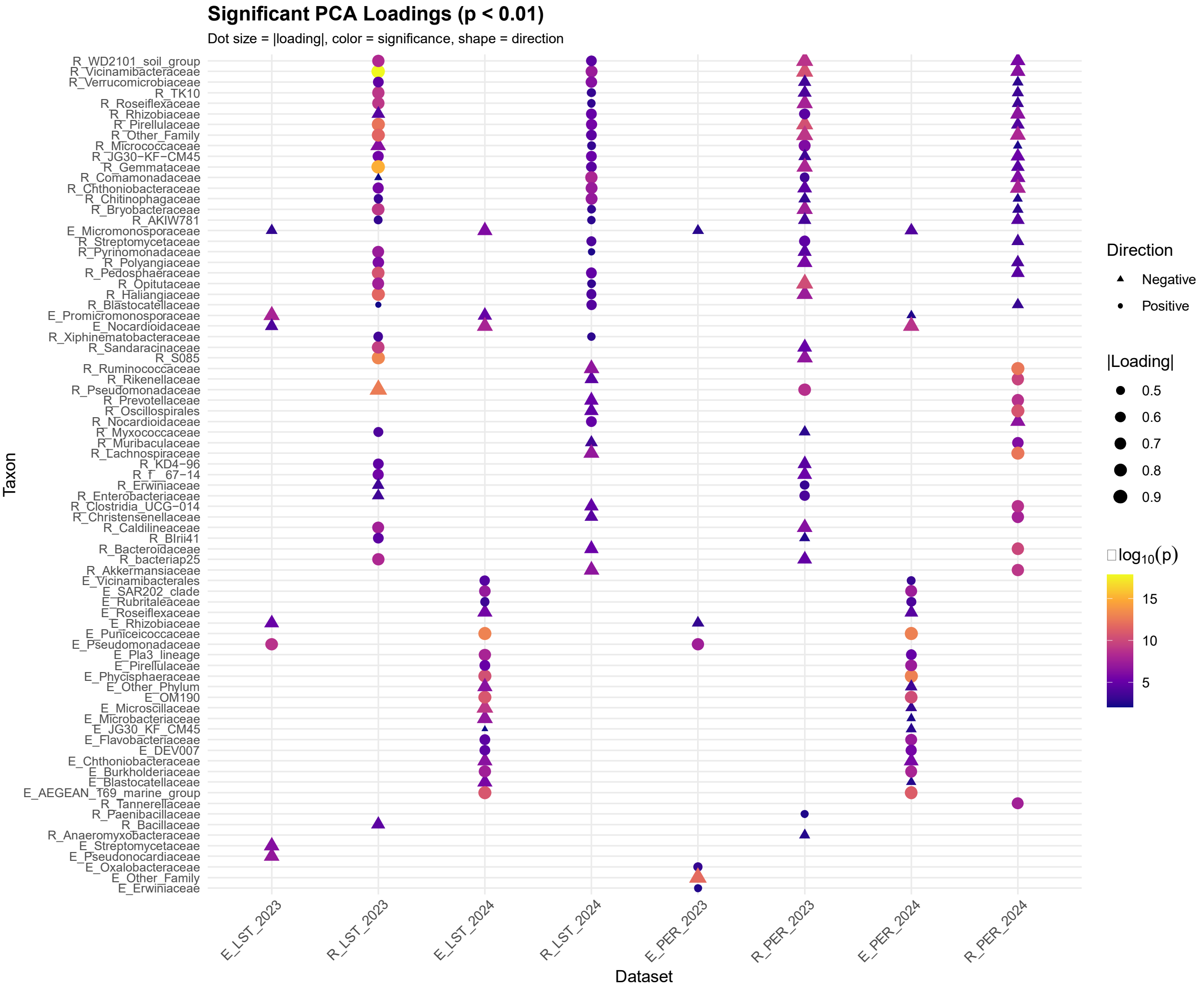

Figure S7. Significant PCA loadings dotplot showing the main taxa contributors in both PC1 and PC2 (*p*<0.01).

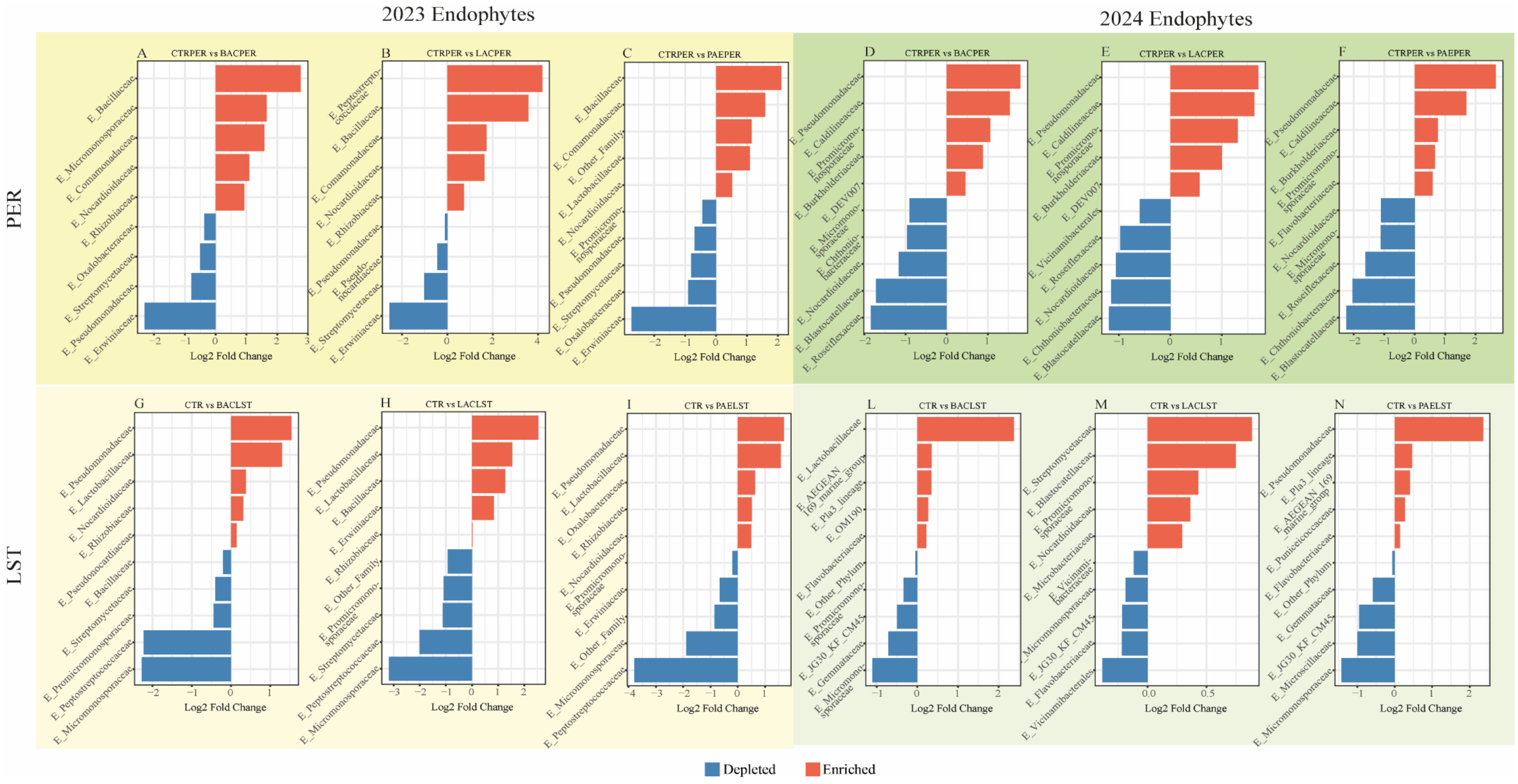

Figure S8: Differential Abundance Analysis (DAA) in endophytes communities occurred in years 2023 and 2024 comparing CTR *vs* treated sunflowers for both PER and LST.

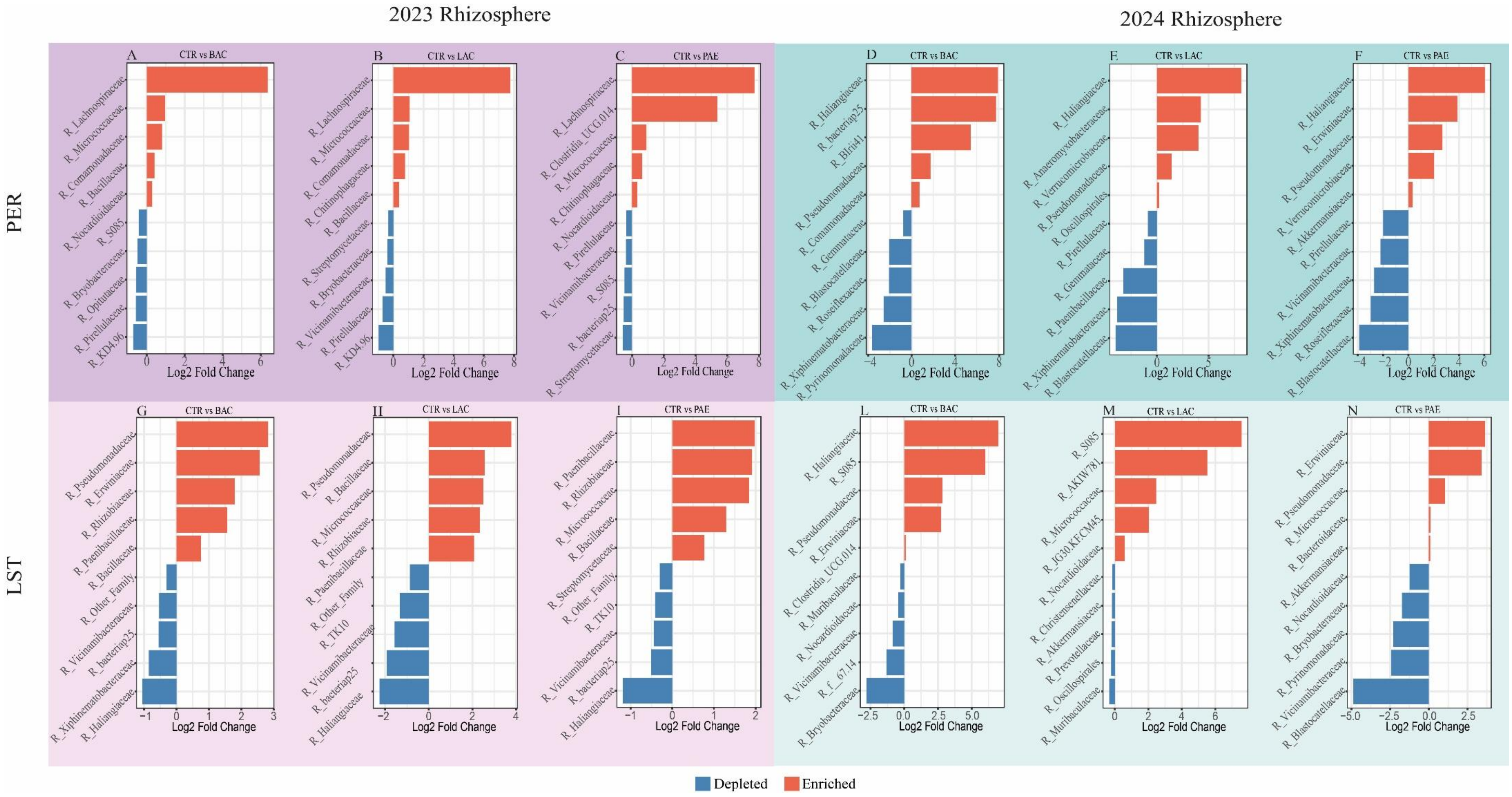

Figure S9: Differential Abundance Analysis (DAA) in rhizosphere communities occurred in years 2023 and 2024 comparing CTR *vs* treated sunflowers for both PER and LST.

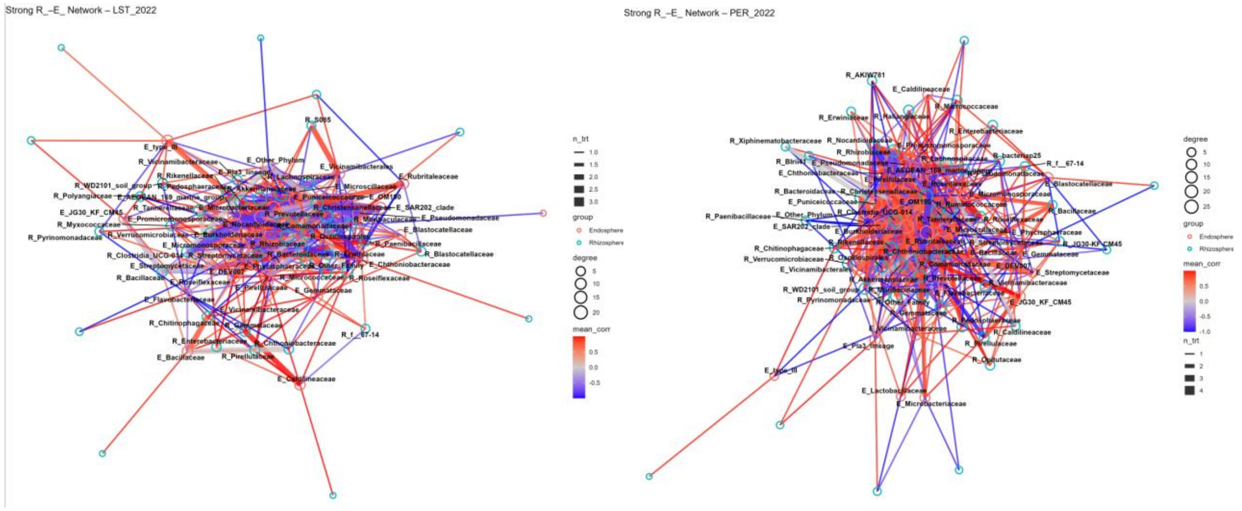

Figure S10. Strong correlation networks between rhizospheric and endophytic microbial communities in A) LST and B) PER varieties during year 2024. Networks report related taxa with $p<0.01$.

Appendix 1: Isolation and characterisation of the PGPB microbial strains

A1.1 Isolation of microbial strains

About 40 microbial strains were isolated from gut content of honeybees, from colony fermented pollen, and from different plant species (such as *Triticum durum*). Isolation matrixes were serially diluted in a 0.85% NaCl solution and plated on TSA (Triptic soy agar), BHI (Brain heart infusion agar) and MRS (de Man, Rogosa and Sharpe agar). Plates were incubated for 5 days at 35 °C, in aerobic conditions. Strains were selectively isolated based on morphology, growth rate and colour. Isolates were characterized with a PCR-dependent fingerprinting technique based on enterobacterial repetitive intergenic consensus (ERIC) sequence using primers ERIC-1 (5'-ATGTAAGCTCCTGGGGATTCAC-3') and ERIC-2 (5'-AAGTAAGTGACTGGGGTGAGCG-3'). Fingerprinting profiles were analyzed with GelCompar II 6.6 (Applied Maths, Kor-trijk, Belgium) using the DICE coefficient and the UPMGA clustering algorithm. DNA amplification of the 16S rRNA gene was performed for samples with an ERIC unique profile, with primers 27f (5'-AGAGTTTGATCCTGGCTCAG-3') and 1492r (5'-GGTTACCTTGTTACGACT-3'), Sanger sequenced and identified on the NCBI database.

A1.2 Characterization of isolated strains as possible PGPB for sunflower growth promotion

The isolated strains were tested for their ability to solubilize MnO_2, PO_4^{3-}, and producing siderophores, which we considered relevant for nutrient mobilization in soil. In addition, we also considered indolacetic acid (IAA) production and antimicrobial activity, as these traits are known to contribute to plant growth promotion and pathogen control.

Manganese oxide solubilization was assessed using Manganese oxide enriched agar, following the protocols proposed by Sanket et al. (2017). Briefly, agar plates added with MnO_2 50 mM were inoculated with the bacterial strains and incubated at 30°C for 48 h. The plates were then checked for the presence of Mn solubilization halos, and its diameter was evaluated as + (weak), ++ (moderate), or +++ (strong).

For the Phosphate (PO_4^{3-}) solubilization assay, each bacterial strain was grown in Nutrient Broth (NB) and incubated at 27 °C in agitation (130 rpm), until reaching an optical density at 600 nm (OD_{600}) of 1.0. A volume of 10 ml from each culture was centrifuged, and the pellet was resuspended in 1 ml of sterile water. 50 µl each were spotted in triplicate Pikovskaya's Agar (HIMEDIA, Germany) plates and incubated at 30 °C for 7 days. The presence of a phosphate solubilization halo was evaluated and the diameter was scored as + (weak), ++ (moderate), or +++ (strong).

Indole-3-Acetic Acid (IAA) production was assessed using a colorimetric spectrophotometric assay using Salkowski reagent methodology (Mayer, 1958). Briefly, microorganisms were individually grown in NB with the addition of L-tryptophan (40 µg/ml) and incubated in agitation (130 rpm) at at 27 °C, in dark condition. After 24, 48 and 72, 10 ml of culture were centrifuged, and 1 ml of the supernatant was mixed with 1 ml of Salkowski reagent (35% perchloric acid and 2% $FeCl_3 \cdot 6H_2O$) and incubated in the dark at 25 °C for 1 hour. A

standard curve was prepared with known IAA concentrations (10, 20, 30, 50, 75, and 100 µg/ml), and the strain absorbance was measured at 550 nm using a spectrophotometer.

Blue Agar CAS Assay was used for siderophore detection following the procedure reported in Louden et al., (2011). Briefly, chrome azurol S(CAS) and hexadecyltrimethylammonium bromide (HDTMA) were used as universal siderophore indicators, according to Schwyn and Neiland (1987) methodology. The siderophore presence was indicated by the color change from blue to yellow, and the halo diameter was scored as + (weak), ++ (moderate), or +++ (strong). The obtained results for each PGPB-associated activity are reported in **Table A1**.

Table A1. Characteristic that differentiates the proposed novel species

Microbial strain tested	Isolation source	MnO_2 solubilization	PO_4^{3-} solubilization	IAA production (µg/ml)	Siderophore (preliminary)
Achromobacter marplatensis S8	Wheat rhizosphere	+	-	NA	-
Apilactobacillus kunkeei DAN39	Honeybee gut	+++	+++	0.00	+++
Azospirillum brasiliense DSMZ 2298	Wheat rhizosphere	+	-	5.50	-
Bacillus aerius SP12	Honeybee pollen	-	+	10.22	-
Bacillus amyloliquefacens P5	Honeybee pollen	-	+	6.15	-
Bacillus amyloliquefacens SP43	Honeybee pollen	-	-	0.00	-
Bacillus licheniformis SP9	Honeybee pollen	-	+	14.07	-
Bacillus subtilis P17	Honeybee pollen	-	-	3.78	-
Bacillus toyonensis S10	Wheat rhizosphere	-	-	7.62	-
Bacillus toyonensis S4	Wheat rhizosphere	-	-	7.09	++
Bacillus toyonensis SP27	Honeybee pollen	-	+	9.93	-
Chryseobacterium lathyri S13	Wheat rhizosphere	-	-	46.39	-
Comamonas sp. S20	Wheat rhizosphere	-	-	0.83	+++
Exiguobacterium sp. C4	Wheat rhizosphere	-	-	2.48	-
Exiguobacterium sp. C5	Wheat rhizosphere	-	-	6.32	-
Paenibacillus alvei PA(A)	Honeybee larvae	-	+	24.82	-
Paenibacillus alvei PA(B)	Honeybee larvae	-	-	8.98	-
Paenibacillus humicus SP29	Soil	-	-	9.99	-
Paenibacillus larvae SP28	Honeybee larvae	-	-	4.55	-
Paenibacillus taichungensis 596 OP	Wheat rhizosphere	-	-	0.47	-
Paenibacillus xylanilithicus SP42	Soil	++	-	4.43	-
Paenibacillus yonginensis F3	Wheat rhizosphere	-	-	7.62	-
Pantoea agglomerans C9	Wheat rhizosphere	-	++	25.12	-
Paraburkholderia phytofirmans PsJN	Wheat rhizosphere	-	-	NA	-
Plantilactobacillus plantarum DAN91	Honeybee gut	+++	+++	NA	-
Plantilactobacillus plantarum LB9	Honeybee gut	+++	+++	0.00	-
Pseudomonas chlororaphis V1	Wheat rhizosphere	-	-	8.63	-
Pseudomonas chlororaphis V2	Wheat rhizosphere	+	+	16.43	-
Pseudomonas chlororaphis V3	Wheat rhizosphere	-	+	14.24	-
Pseudomonas mosselii S12	Wheat rhizosphere	+	-	9.57	-
Pseudomonas plecoglossicida G2	Wheat rhizosphere	-	-	3.84	-
Pseudomonas plecoglossicida G3	Wheat rhizosphere	-	-	3.19	-
Pseudomonas plecoglossicida S1	Wheat rhizosphere	-	-	30.67	-
Pseudomonas putida C7	Wheat rhizosphere	-	++	7.57	-
Pseudomonas putida S6	Wheat rhizosphere	-	++	14.36	-
Pseudomonas reidholzensis S22	Wheat rhizosphere	-	-	1.77	-
Pseudomonas tumulicola G1	Wheat rhizosphere	-	-	6.44	+
Rhizobium nepotum S3	Wheat rhizosphere	-	-	8.75	-
Sphingobacterium athyrii S14	Wheat rhizosphere	+	-	2.96	-
Sphingobacterium athyrii S17	Wheat rhizosphere	-	-	15.72	+
Sphingobacterium canadense M4	Wheat rhizosphere	+	-	9.04	+++

Sphingobacterium canadense M4B	Wheat rhizosphere	-	-	6.62	+++
Sphingobacterium canadense S19	Wheat rhizosphere	-	-	5.32	-
Sphingobacterium canadense S5	Wheat rhizosphere	-	-	2.13	-
Stenotrophomonas pavanii S7	Wheat rhizosphere	-	-	9.69	+
Stenotrophomonas rhizophila C2	Wheat rhizosphere	-	-	6.21	-
Stenotrophomonas sp. C3	Wheat rhizosphere	-	+	82.57	-
Xanthomonas sp. M3	Fruit plant (Malus)	-	-	6.50	-
Pseudomonas sp. C6	Fruit plant (Cherry)	-	-	0.71	-
Pseudomonas sp. C8	Fruit plant (Cherry)	-	-	8.69	-
Pseudomonas sp. M1	Fruit plant (Malus)	-	-	6.03	-
Pseudomonas sp. S16	Aromatic plant (Salvia)	-	-	4.55	-
Pseudomonas sp. S18	Aromatic plant (Salvia)	-	-	13.24	-
Pseudomonas sp. S21	Aromatic plant (Salvia)	+	-	2.07	-
Pseudomonas sp. S25	Aromatic plant (Salvia)	-	-	NA	-

The agar well diffusion assay was carried out as described by Cintas et al. (1995) on soft agar inoculated with indicator strains. The indicator strains used are reported in Figure A1, with the exclusion of *Clostridium*, *Enterococcus*, *Listeria monocitogenes* and *Streptococcus*, and the same isolated strains (all against each other). The antimicrobial activity was evaluated, and the halo diameter was scored as + (weak), ++ (moderate), +++ (strong), or very strong (++++). The obtained results were reported in **Figure A1.**

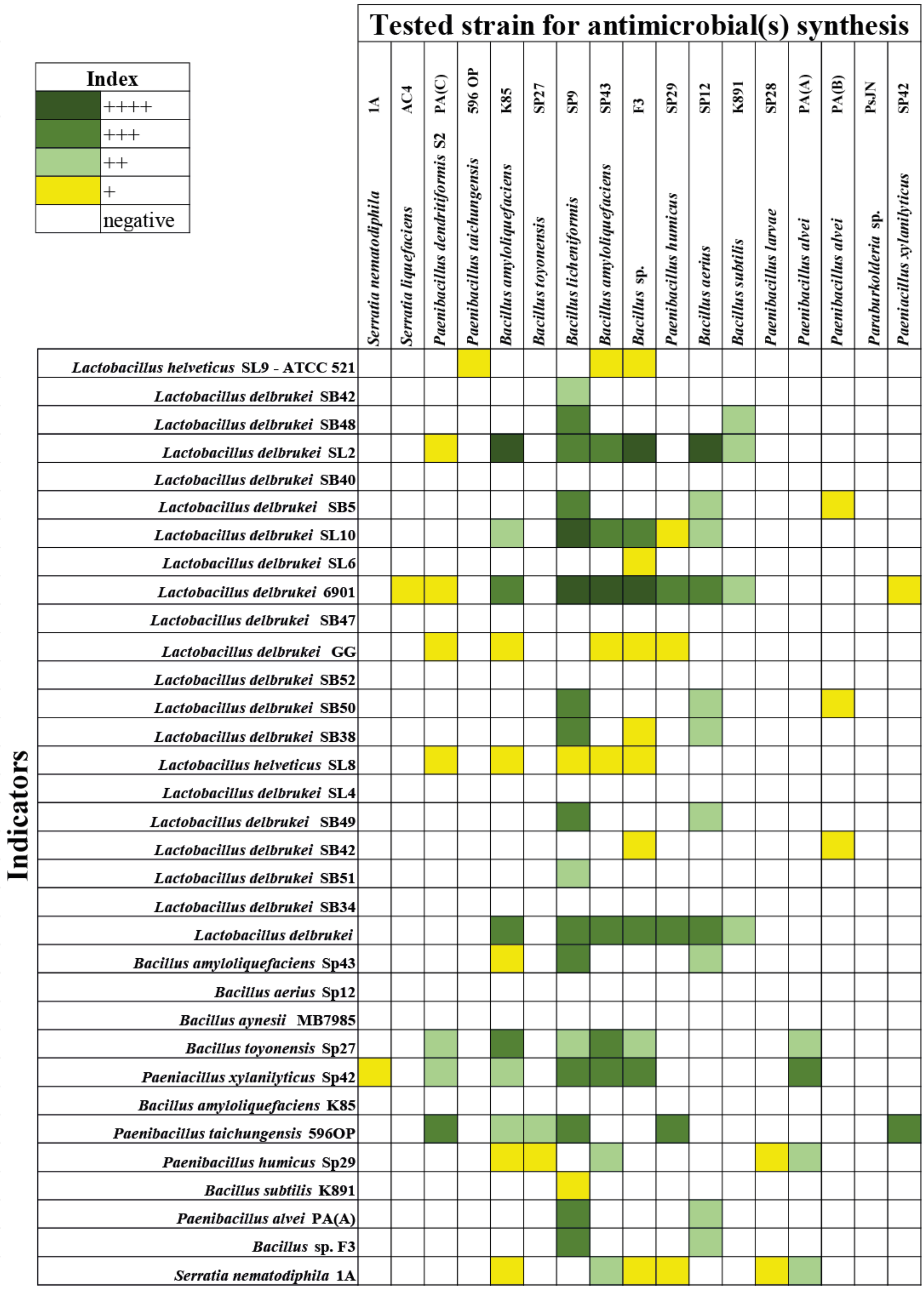

Figure A1. Agar well diffusion assay results. White cells indicate no- inhibition effects.

A1.3 Bibliography

Cintas, L.M., Rodriguez, J.M., Fernandez, M.F., Sletten, K., Nes, I.F., Hernandez, P.E., Holo, H., 1995. Isola tion and characterization of pediocin L50, a new bacteriocin from pediococcus acidilactici with a broad inhibitory spectrum. Appl. Environ. Microbiol. 61 (7), 2643. doi:10.1128/aem.61.7.2643-2648.1995.

Louden, B.C., Haarmann, D., Lynne, A.M., 2011. Use of blue agar CAS assay for siderophore detection. JMBE 12 (1), 51–53. doi:10.1128/jmbe.v12i1.249.

Mayer, A.M., 1958. Determination of indole acetic acid by the Salkowsky reaction. Nat. doi:10.1038/1821670a0.

Sanket, A.S., Ghosh, S., Sahoo, R., Nayak, S., Das, A.P., 2017. Molecular identification of acidophilic manganese (Mn)-solubilizing bacteria from mining effluents and their application in mineral beneficiation. Geomicrobiol. J. 34 (1), 71–80. doi:10.1080/01490451.2016.1141340.

Schwyn, B., Neilands, J.B., 1987. Universal chemical assay for the detection and determination of siderophores. Anal. Biochem. 160, 47–56. doi:10.1016/0003-2697(87)90612-9.

Appendix 2: the main shift highlighted in the PCA analysis

The highest impact was observed in the LST rhizosphere in 2023 and in the LST endosphere in 2024, indicating strong treatment-related shifts in microbial communities (**Figures S1A-S1D** and **S2A- S2D**). Overall, the PCA results highlight clear influences of variety, year, and compartment on the sunflower-associated microbiota. LST exhibited more pronounced treatment-driven shifts, particularly in the rhizosphere. At the same time, PER showed slight and specific variations, with the endosphere being more responsive in 2023 and both compartments showing clear structure in 2024.

In details, in the LST endosphere (2023, **Figure S1A**), E_Pseudonocardiaceae (loading= -0.75; $p<0.01$), E_Streptomycetaceae (loading= -0.72; $p<0.01$), and E_Nocardioidaceae (loading= -0.60; $p<0.01$) showed strong negative contributions to both PC1 and PC2 and when treatments are applied. When 2024 was analysed, in the LST endosphere (2024, **Figure S1C**), key loading taxa included E_Blastocatellaceae (loading= -0.70; $p<0.01$), E_ Chthoniobacteraceae (loading= -0.73; $p<0.01$), and E_Flavobacteriaceae (loading= 0.64; $p<0.01$), suggesting enrichment of beneficial plant-associated microbes.

In the LST rhizosphere (2023, **Figure S1B**), dominant negative contributors included R_Bacillaceae (loading= -0.64; $p<0.01$), R_Comamonadaceae (loading= -0.43; $p<0.01$), and R_Pseudomonadaceae (loading= -0.89; $p<0.01$), reflecting their roles in differentiating microbial profiles among treatments. Moreover, R_Gemmataceae (loading= 0.92; $p<0.01$), R_Vicinamibacteraceae (loading= 0.95; $p<0.01$), and R_Pirellulaceae (loading= 0.89; $p<0.01$) had a strong positive contribution in both PC1 and PC2. In 2024, the LST rhizosphere (2024, **Figure S1D**) was mainly influenced by R_Prevotellaceae (loading= -0.67; $p<0.01$), R_Chitinophagaceae (loading= 0.75; $p<0.01$), and R_Blastocatellaceae (loading= 0.61; $p<0.01$), driving separation along both principal components.

For the PER variety, community structures also varied across years and compartments (rhizosphere or endosphere), although shifts were often minor than in LST. In the PER endosphere (2023, **Figure S2A**) (PC1 = 25.5%, PC2 = 19.4%), major contributing taxa included E_Pseudomonadaceae (loading= 0.79; $p<0.01$), E_Oxalobacteraceae (loading= 0.54; $p<0.01$), and E_Rhizobiaceae (loading= -0.55; $p<0.01$), consistent with a treatm ent-driven modulation of the endophytic microbiota. On the other hand, in PER 2024, the endosphere was primarily shaped by E_Micromonosporaceae (loading= -0.61; $p<0.01$), E_Flavobacteriaceae (loading= 0.75; $p<0.01$), and E_Promicromonosporaceae (loading= -0.46; $p<0.01$) (**Figure S2C**),

The PER rhizosphere (2023, **Figure S2B**) showed differentiation mainly due to R_Pseudomonadaceae (loading= 0.83; $p<0.01$), R_Bacillaceae (loading= 0.40; $p<0.05$), and R_Comamonadaceae (loading= 0.58; $p<0.01$). In 2024, the rhizosphere displayed different separation associated with R_Lachnosiraceae (loading= 0.88; $p<0.01$), R_Chitinophagaceae (loading= -0.47; $p<0.01$), and R_Ruminococcaceae (loading= 0.88; $p<0.01$) (**Figure S2D**).

Appendix 3: Differential Abundance Analysis among the different PGPB treatment during the two seasons (2023-2024)

Considering DAA in the specific treatment BAC, LAC and PAE, significant differences were found when rhizosphere and endophytes communities were compared within the two sunflower varieties (PER *vs* LST). In 2023, BAC administration significantly increased the abundance of R_Opitutaceae (LFC=1.8, p<0.01), E_Nocardioidaceae (LFC=1.09, p<0.01), and E_Promicromonosporaceae (LFC=1.19, p<0.5), while decreased the abundance of some taxa such as R_Pedosphaeraceae (LFC=-2.15, p<0.01), R_Lachnospiraceae (LFC=-2.48, p<0.01), and E_Erwiniaceae (LFC= -0.83, p<0.05) in PER when compared with LST (**Figure A2A**). LAC administration modified the PER rizosphere and endophyte communities highlighting abundance differences in taxa such as R_Opitutaceae (LFC=2.36, p<0.01), R_Bacillaceae (LFC=-1.34, p<0.01), R_Enterobacteriaceae (LFC=-2.41, p<0.01), E_Nocardioidaceae (LFC=2.14, p<0.01), and E_Erwiniaceae (LFC=-1.63, p<0.05) when compared to LST (**Figure A2B**). Conversely, PAE inoculation induced the lowest number of significant variations in the microbial communities between the two varieties, with an increasing of R_Opitutaceae (LFC=1.87, p<0.01), and a decreasing of R_Pedosphaeraceae (LFC=-1.27, p<0.05) (**Figure A2C**).

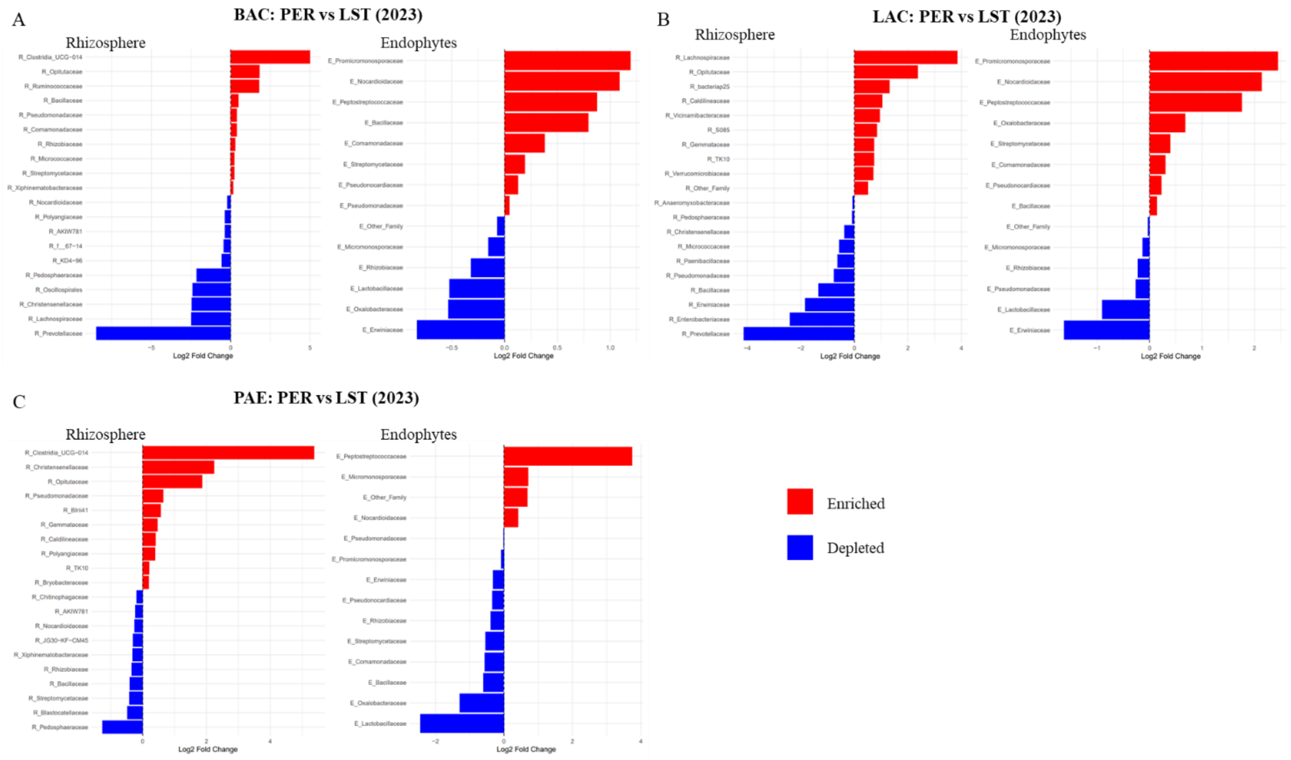

Figure A2. Differential Abundance Analysis (DAA) in rizosphere and endophytes communities occurred in year 2023 comparing PER *vs* LST treated with A) BAC, B) LAC, and C) PAE.

In 2024, upon BAC administration, variations in R_Rhizobiaceae (LFC=1.20, p<0.01), R_Prevotellaceae (LFC=-0.31, p<0.01), unexplored taxa such as E_SAR202_clade (LFC=-0.7, p<0.01) and E_type_III (LFC=-1.62, p<0.05), and Streptomycetacea (LFC=1.08, p<0.05) occurred in PER when compared to LST (**Figure A3A**). Moreover, fewer significant variations were detected in LAC and PAE-treated sunflowers in PER vs LST, also compared to 2023. Specifically, LAC treatment modified the abundance of R_Haliangiaceae

(LFC=3.94, *p*<0.05), unexplored taxa as R_S085 (LFC=-2.09, *p*<0.05) and R_JG30-KF-CM45 (LFC=-2.16, *p*<0.05), and E_Blastocatellaceae (LFC=-1.09, *p*<0.05) in PER when compared to LST (**Figure A3B**). Finally, PAE decreased microbial load of R_Muribaculaceae (LFC=-0.53, *p*<0.05), R_Ruminococcaceae (LFC=-0.21, *p*<0.05) and E_OM190 (LFC=-0.66, *p*<0.05), while E_Microbacteriaceae (LFC=0.94, *p*<0.01) were found to increase in PER respect to LST (**Figure A3C**).

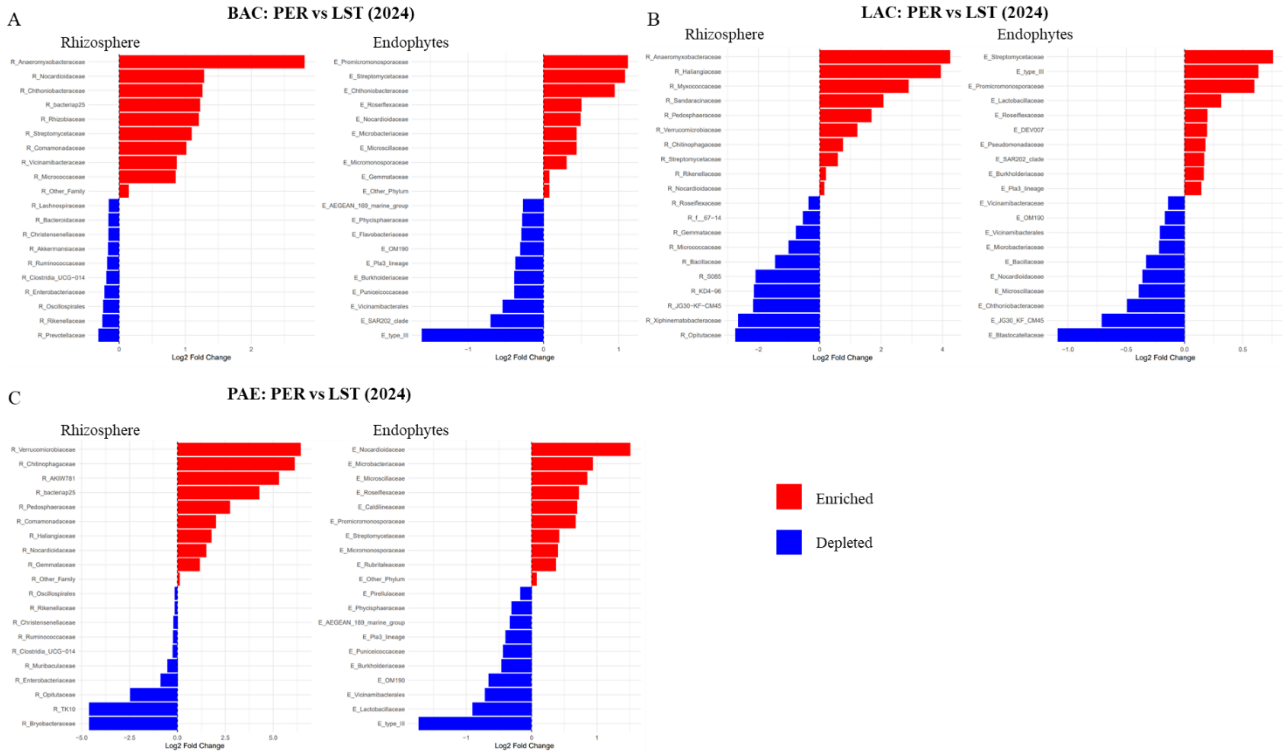

Figure A3. Differential Abundance Analysis (DAA) in rizosphere and endophytes communities occurred in year 2024 comparing PER *vs* LST treated with A) BAC, B) LAC, and C) PAE.

Study limitations

This two-year field study was conducted in an area that, over the past decade, has typically experienced prolonged droughts periods or below average rainfalls. In contrast, the 2024 season was marked by flooding and extended periods of unusually intense precipitation across the trial plots. These hydroclimatic anomalies likely confounded treatment effects and reduced comparability across years. Waterlogging alters soil oxygen availability and redox conditions, modifies nutrient dynamics, and affects sunflower root physiology and exudation profiles. Such shifts can hinder root colonization or colonization by plant growth-promoting bacteria, attenuating the magnitude of microbiome and agronomic responses. Accordingly, the modest PGPB treatment responses observed in 2024 should be interpreted in the context of an exceptional season rather than as evidence of limited efficacy under typical conditions.

Future work should incorporate multi-site and multi-year replication spanning both dry and wet hydrological regimes, continuous monitoring of soil moisture, and strain-specific tracking of inoculant persistence. Designs that include controlled irrigation or rain-exclusion shelters, along with quantification of absolute abundances and activity-based readouts, would help disentangle true treatment effects from weather-driven variability and improve the generalizability of findings.

Supplementary File 1: Content descriptions

The supplementary file 1 contains all the data from NGS and statistical analysis for:

Sheet 1: "*LST_rhizo and endophytes*": NGS relative abundance of taxa detected in rhizosphere and endophytes microbial community in LST907 variety for both 2023 and 2024.

Sheet 2: "*PER_rhizo and endophytes*": NGS relative abundance of taxa detected in rhizosphere and endophytes microbial community in Peredovik variety for both 2023 and 2024.

Sheet 3: "*PCA_ significant loadings*": significant contributors' taxa ($p<0.01$) among rhizosphere and endophytes microbial community for both varieties and years.

Sheet 4: "*Sankey_plot_correlation*": Sankey plot correlation data

Sheet 5: "*Correlation_R-E*": statistical output for correlation analysis among rhizosphere and endophytes microbial community.

Sheet 6: "*Recurrent hub*": most recurrent correlation between rhizosphere and endophytes microbial community.

Sheet 7: "*Node and Degree*": node and degree used for network analysis

Supplementary File 2: Content descriptions

The supplementary file 2 illustrates the inferred summary outputs related to PGPB effects on the sunflower root microbiota.

Sheet 1: “Families varying under PGPB”
List of microbial families that vary across PGPB treatments (24 families). The column is titled “Families varying under PGPB treatments.”

Sheet 2: “Functionality computation”
Table summarizing trait-level associations between experimental conditions, PGPB functional traits, and microbial families.

Columns of **Sheet 2**:

Experimental conditions: combinations of variety, year, and treatment group (for example LST_2023_BAC, PER_2024_PAE).

PGPB trait considered: functional categories as labeled in the sheet (ACC deaminasi, Biocontrollo/ISR, Degradazione cellulosa, EPS/Biofilm, Fissazione N2, Fitormoni (IAA ecc.), Siderofori, Solubilizzazione P).

Microbial family: taxonomic family involved.

Sign: direction of the association or effect (−1 negative, 0 neutral, 1 positive).

Weight: relative weight assigned to each entry.

Trait_score: discrete score summarizing trait relevance per entry.

Score_weight_log2_FC: composite metric that combines score, weight, and log2 fold change for interpretation of effect magnitude.
